# Evolutionary Rate Covariation Identifies New Members of a Protein Network Required for *Drosophila melanogaster* Female Post-Mating Responses

**DOI:** 10.1371/journal.pgen.1004108

**Published:** 2014-01-16

**Authors:** Geoffrey D. Findlay, Jessica L. Sitnik, Wenke Wang, Charles F. Aquadro, Nathan L. Clark, Mariana F. Wolfner

**Affiliations:** 1 Department of Molecular Biology and Genetics, Cornell University, Ithaca, New York, United States of America; 2 Department of Computational and Systems Biology, University of Pittsburgh, Pittsburgh, Pennsylvania, United States of America; University of Michigan, United States of America

## Abstract

Seminal fluid proteins transferred from males to females during copulation are required for full fertility and can exert dramatic effects on female physiology and behavior. In *Drosophila melanogaster*, the seminal protein sex peptide (SP) affects mated females by increasing egg production and decreasing receptivity to courtship. These behavioral changes persist for several days because SP binds to sperm that are stored in the female. SP is then gradually released, allowing it to interact with its female-expressed receptor. The binding of SP to sperm requires five additional seminal proteins, which act together in a network. Hundreds of uncharacterized male and female proteins have been identified in this species, but individually screening each protein for network function would present a logistical challenge. To prioritize the screening of these proteins for involvement in the SP network, we used a comparative genomic method to identify candidate proteins whose evolutionary rates across the *Drosophila* phylogeny co-vary with those of the SP network proteins. Subsequent functional testing of 18 co-varying candidates by RNA interference identified three male seminal proteins and three female reproductive tract proteins that are each required for the long-term persistence of SP responses in females. Molecular genetic analysis showed the three new male proteins are required for the transfer of other network proteins to females and for SP to become bound to sperm that are stored in mated females. The three female proteins, in contrast, act downstream of SP binding and sperm storage. These findings expand the number of seminal proteins required for SP's actions in the female and show that multiple female proteins are necessary for the SP response. Furthermore, our functional analyses demonstrate that evolutionary rate covariation is a valuable predictive tool for identifying candidate members of interacting protein networks.

## Author Summary

Reproduction requires more than a sperm and an egg. In animals with internal fertilization, other proteins in the seminal fluid and the female are essential for full fertility. Although hundreds of such reproductive proteins are known, our ability to understand how they interact remains limited. In this study, we investigated whether shared patterns of protein sequence evolution were predictive of functional interactions by focusing on a small network of proteins that control fertility and female post-mating behavior in the fruit fly, *Drosophila melanogaster*. We first showed that the six proteins already known to act in this network display correlated patterns of evolution across the *Drosophila* phylogeny. We then screened hundreds of otherwise uncharacterized male and female reproductive proteins and identified those with patterns of evolution most similar to those of the known network proteins. We tested each of these candidate genes and found six new network members that are each required for long-term fertility. Using molecular genetics, we also observed that the steps in the network at which these new proteins act are consistent with their strongest evolutionary correlations. Our results suggest that patterns of coevolution may be broadly useful for predicting protein interactions in a variety of biological processes.

## Introduction

Sexual reproduction is a fundamental biological process by which many eukaryotic organisms transmit their genetic material to the next generation. While the end result of a successful mating is the fusion of the gametes, other molecular interactions must occur to allow this fusion. In internally fertilizing animals, males transfer to females not only sperm, but also a suite of seminal fluid proteins (Sfps) that are essential for reproductive success. Across diverse taxa, Sfps are required for: the mobilization of sperm and their storage within the female; increasing the reproductive capacity of the female; affecting the outcome of sperm competition between multiple males; and, facilitating the union of the gametes [reviewed in 1]. In insects, Sfps also alter female behaviors and physiology [2]. Effects of Sfps can be caused by interactions between specific Sfps, between Sfps and proteins on the sperm, and between Sfps and proteins native to the female reproductive tract. Thus, characterizing the functions and interactions of Sfps is important for understanding how the sexes together ensure the successful production of progeny.

Post-mating changes in physiology and behavior induced by Sfps have been extensively characterized in *Drosophila melanogaster* [2], [3]. In response to the receipt of Sfps, females produce, ovulate and lay eggs [4]–[6]; store sperm in specialized storage organs [7]–[10]; show altered immune responses [11], [12]; undergo changes in sleeping, feeding and excretion behavior [13]–[16]; and, become refractory to male courtship [17], [18]. Several of these behavioral changes – egg production, sperm storage and release, and refractoriness to remating – persist in females for several days after mating and have thus been termed the long-term response [19]–[21]. The proximate cause of these changes is a short (36 amino acid) seminal protein called sex peptide (SP) [17], [18]. While most Sfps are no longer detectable in females several hours after mating [22], SP persists in females for days by binding to stored sperm [19]. Gradually, the C-terminal portion of the peptide is proteolytically cleaved to release it from sperm into the female reproductive tract [19]. This C-terminal portion of SP can then signal through its receptor, sex peptide receptor (SPR), which prolongs at least some behavioral changes in the female [23]–[26]. Indeed, SP cleavage is required for the protein to affect female behavior for more than one day [19] and for sperm to be released efficiently from storage [27].

We have previously used RNA interference (RNAi) or gene knockout lines to test 32 Sfps for function in the SP-mediated long-term response [4], [7], [10], [20], [28], [29]. These studies identified five proteins that are required for SP to function over the long term in mated females: two C-type lectins, CG1652 and CG1656; a serine protease homolog, CG9997; a cysteine-rich secretory protein, CG17575; and, a serine protease, seminase (CG10586). These proteins act in a network in which each member is required for SP to become bound to sperm [21], [28]. Loss of any network protein causes an early resumption of female receptivity to remating and a decrease in long-term fecundity. Such loss also impairs the release of sperm from the seminal receptacle in the days following mating [27]. Specific members of the network act interdependently on one another. For example, males that do not produce CG9997 are unable to transfer CG1652 and CG1656 to the female, while CG1652 and CG1656 are required to slow the rate at which CG9997 is processed in the female. Thus, while SP-SPR signaling is the proximate cause of the female post-mating response, several additional Sfps are required for this signaling to persist over the long term. We refer to this set of seven proteins as the SP network.

While genomic and proteomic analyses in *D. melanogaster* have identified hundreds of proteins from sperm [30], [31], seminal fluid [32]–[35], and the female sperm storage organs [36]–[40], we know of few examples of how these proteins interact to cause the dramatic post-mating phenotypes observed in females [21], [26], [28]. Biochemical approaches to identify interacting proteins are challenging due to the small amount of protein per fly, and exhaustive genetic screening of each known reproductive protein would be laborious. Here, we demonstrate a successful effort to prioritize male and female proteins for functional testing by examining covariation in their rates of evolution among species.

Evolutionary Rate Covariation (ERC) is a new metric that bioinformatically infers functional relationships between proteins based solely on their evolutionary rates across an array of species [41]. ERC operates from the hypothesis that functionally related proteins will experience correlated rate changes, because forces governing protein evolutionary rate are expected to influence entire pathways simultaneously. Evolutionary rate depends on several factors including a protein's expression level, its essentiality, and its interactions with other proteins [42]–[49]. Pathway-wide fluctuation in each of these factors has been associated with correlated rate changes (i.e., ERC) between functionally related proteins [41], [50]–[53].

In practice, an ERC value is calculated by computing the correlation between the rates of change of two proteins across all branches of a phylogeny. ERC values range from 1 to −1 for a perfect positive or negative correlation, respectively, with the genome-wide ERC distribution between all protein pairs centered at zero [41]. Functionally related pairs of proteins have been observed to have more positive ERC values in taxa as diverse as eubacteria, fungi, invertebrates and mammals [41], [50], [51], [54]–[58]. This finding holds for proteins that share physical or genetic interactions and proteins that are found in common complexes or metabolic pathways [41], [59]. Generally, a high ERC value is best interpreted as a potential functional link, which could have resulted from a common evolutionary force acting on both proteins. Accordingly, we can infer that proteins with correlated rates may be functionally related.

ERC and related methods have primarily been used to study proteins that are already known to interact functionally or physically; the use of such methods for functional prediction is only now starting to emerge [60]. We tested the utility of applying ERC prospectively by examining proteins required for *Drosophila* SP function. Because proper function of the SP network is essential for fertility, we reasoned that members of this network could have experienced shared evolutionary selective pressures over time and might thus show patterns of ERC across the phylogeny of sequenced *Drosophila* species [61]. To test this hypothesis, we created an ERC dataset specific to *Drosophila*. This analysis revealed significant levels of ERC between known members of the SP network. We then screened for new members of this network by searching for elevated ERC between known network proteins and sets of uncharacterized Sfps and female reproductive proteins. RNAi tests of 18 top candidates revealed three female and three male proteins required for network function. Through molecular genetic analysis, we placed five of these proteins into specific positions in the SP network, and we observed that the steps in the network in which these new proteins act are largely consistent with their evolutionary correlations. Our results demonstrate that signatures of ERC can be used prospectively to predict members of a protein network, suggesting that this method may be broadly applicable for identifying novel protein interactions.

## Results

### Proteins in the SP network show correlated evolutionary rate variation

We first calculated Evolutionary Rate Covariation (ERC) values for all pairs of orthologous proteins (reproductive and otherwise) from 12 *Drosophila* species. Briefly, we assembled orthologous protein sequences for each gene from each species for which they were available, resulting in 11,100 multiple alignments. For each pair of alignments, we calculated the correlation coefficient between their branch-specific evolutionary rates (see Methods and Figure S1). The resulting ERC values ranged from −1 to 1 and reflect the degree to which evolutionary rates correlate for any particular pair of proteins. Typically, ERC values between functionally related protein pairs are elevated compared to unrelated pairs [55]. We observed this same pattern for the seven previously known members of the *Drosophila* SP network. ERC values calculated for all possible pairs of these seven proteins had a mean of 0.3115, compared to the proteome-wide mean of 0.0019 (Figure S1A). The highly significant elevation between SP network proteins (permutation *p* = 0.000154) suggests that ERC could be used to predict additional SP network proteins. However, since proteins that are expressed at similar levels or in similar patterns can also show correlated evolution [43], we also tested whether reproductive proteins as a class had elevated ERC values. To do so, we examined a set of 664 proteins found in seminal fluid, sperm, or female sperm storage organs (see Methods and Figure S1A; we refer to these proteins below as “reproductive” but note that some are also expressed in non-reproductive tissues and could thus have other functions). The mean ERC value between all reproductive proteins was 0.0326, a highly significant elevation for sets of this size (permutation *p*<0.0001). This elevation could be driven by direct functional relationships and/or more indirect relationships such as expression patterns [41].

To control for this elevation in ERC across all reproductive proteins when evaluating correlations between individual pairs of proteins, we factored out the broad relationship between them. To do so, we recalculated ERC using only the 664 reproductive proteins to estimate the background rate of evolution, instead of all 11,100 proteins (see Methods and Figure S1B). After this adjustment, the mean pairwise ERC between all proteins in the reproductive set fell to 0.0047. By contrast, the mean correlation between the seven known SP network proteins remained significantly elevated (mean = 0.2806; permutation *p* = 0.001002). These results suggest that while shared patterns of expression or function can cause a significant increase in ERC, a much stronger signal is shared by the specific set of proteins that act together in the SP network.

**Figure 1:**
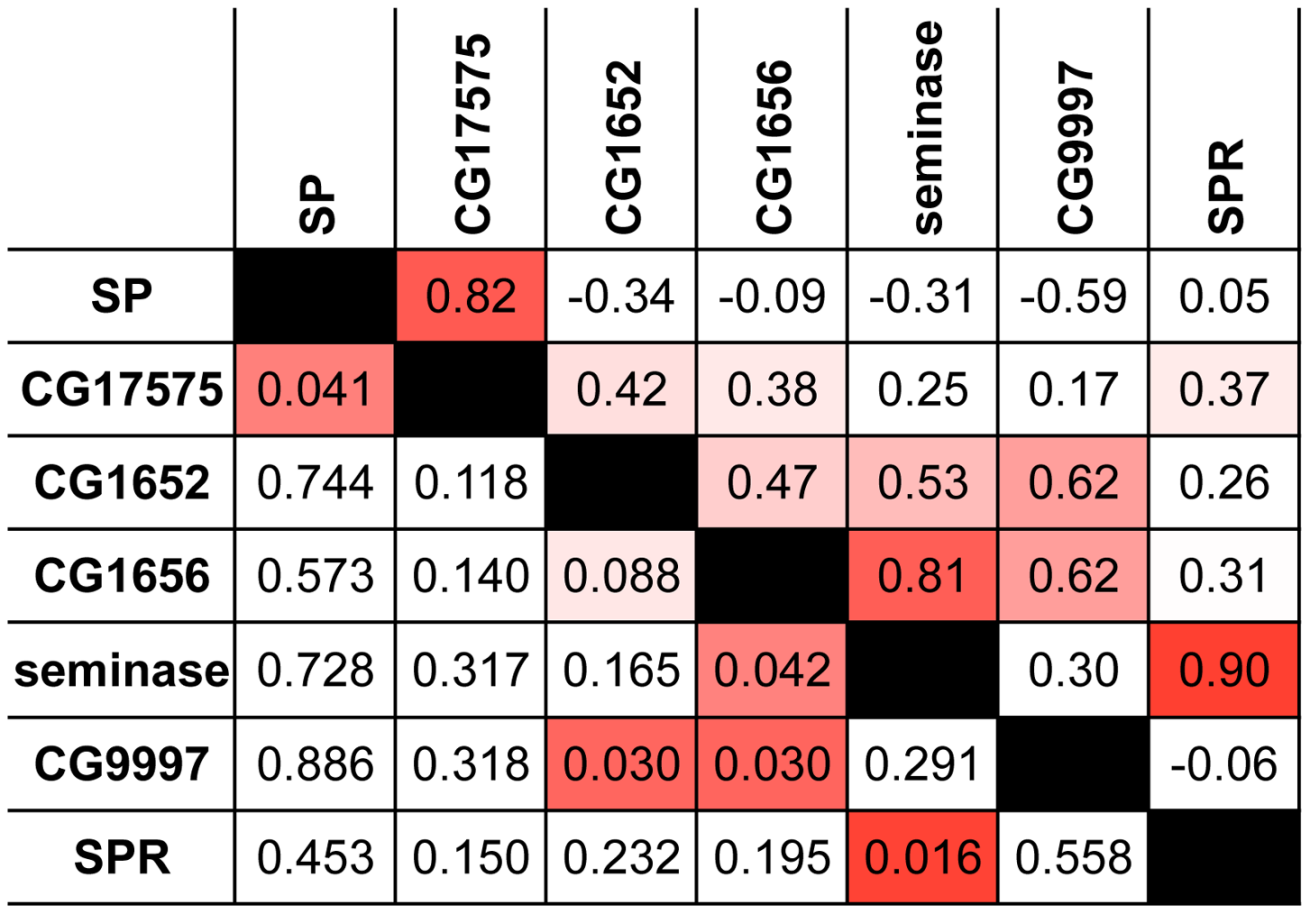
Proteins in the SP network show a significantly elevated signature of ERC. This pairwise matrix shows ERC values (above diagonal) and their corresponding empirical *p*-values (below diagonal) between the seven known members of the SP network. Red shading indicates correlations with empirical *p*<0.05; more intense shading indicates a stronger correlation.

Several of the strongest pairwise correlations between known members of the SP network were found between proteins with recognized genetic interactions. For example, males that do not produce network protein CG9997 are unable to transfer CG1652 and CG1656 to females during mating [21]. These pairs of proteins show ERC values in the top 5 percent of all pairwise correlations (CG9997-CG1652: *r* = 0.62, empirical *p* = 0.03; CG9997-CG1656: *r* = 0.62, empirical *p* = 0.03; Figure 1). In other instances, we did not observe strong correlations between proteins that might be expected to coevolve, such as SP and SPR. However, this particular lack of correlation may be explained by the fact that SPR has additional, non-reproductive ligands besides SP [62], [63], which may constrain its evolution. Nonetheless, the overall signature of correlated evolution throughout the SP network, the high proportion of positive pairwise correlations in the group (i.e., 16 of the 21 pairwise correlations in Table 1 are positive), and the significant correlations between specific group members suggest that members of the SP network show significant levels of evolutionary rate covariation.

**Table 1:** Candidates identified by ERC and tested for effects on 4-day remating receptivity.

Gene Name	Predicted functional class	Expression pattern*	Significant ERC results	Amount of knockdown	4-Day Receptivity Assay
*CG30433*	C-type lectin	male AG	CG17575: p = 0.025CG1652: p = 0.037SP: p = 0.042	near-complete	KD: 7/33cont: 3/30p = 0.31
*CG11037*	chymotrypsin-like	male AG	CG9997: p = 0.015CG1652: p = 0.029	partial	KD: 1/30cont: 0/29p = 1.00
*CG11977*	CRISP	male AG	CG9997: p = 0.011CG1652: p = 0.049	near-complete	KD: 2/36cont: 3/39p = 1.00
*CG14034*	lipase	male AG	CG1652: p = 0.029CG9997: p = 0.043	near-complete	KD: 9/30cont: 8/45p = 0.27
***CG14061 (aqrs)***	serine protease homolog	male AG	CG1652: p = 0.0015CG9997: p = 0.02CG1656: p = 0.035	near-complete	**KD: 27/29** **cont: 0/29** **p<0.0001**
*CG2975*	galactosyltransferase	male AG, crop	CG17575: p = 0.003SP: p = 0.03	complete	KD: 0/34cont: 5/28p = 0.015
***CG30488 (antr)***	CRISP	male AG	CG9997: p = 0.009	complete	**KD: 29/32** **cont: 3/29** **p<0.0001**
*CG42326*	unknown	male AG, head, eye	CG9997: p = 0.015CG1652: p = 0.033	near-complete	KD: 2/33cont: 3/31p = 0.67
***CG12558 (intr)***	serine protease homolog	male AG	CG9997: p = 0.007	near-complete	**KD: 11/14** **cont: 3/16** **p = 0.0027**
*CG42564*	CRISP	male AG	CG9997: p = 0.003	near-complete	KD: 4/32cont: 2/33p = 0.43
*CG8420*	unknown	male AG	CG1652: p = 0.007	partial	KD: 1/33cont: 2/33p = 1.00
*CG13077*	cytochrome b561	female ST, eye, head	CG1656: p = 0.009	near-complete	KD: 3/33cont: 9/33p = 0.11
*CG16713*	Kunitz protease inhibitor	female ST, FB, hindgut, head, eye	CG1652: p = 0.009CG17575: p = 0.022CG9997: p = 0.042	none detected	n/a
*CG3097*	peptidase M14	female ST, hindgut, crop	CG9997: p = 0.0007CG1652: p = 0.011	complete	KD: 1/36cont: 2/39p = 1.00
***CG3239 (frma)***	protease/neprilysin	female ST, FB, head, heart	CG17575: p = 0.008	partial	**KD: 21/30** **cont: 1/30** **p<0.0001**
*CG4302*	UDP-glucosyltransferase	female ST, MT, FB, eye, TG, head, brain	CG1656: p = 0.002CG9997: p = 0.021	none detected	n/a
*CG6910*	inositol oxygenase	female ST, heart, FB	CG1656: p = 0.007CG17575: p = 0.047	partial	KD: 1/30cont: 4/31p = 0.35
*CG8586*	chymotrypsin-like	female ST, head, FB, eye, crop, heart	CG1656: p = 0.008SP: p = 0.022CG17575: p = 0.042	none detected	n/a
*Mtp*	phosphatidylcholine transpoter	female ST, FB, head, heart, eye, brain, TG, crop	CG1652: p = 0.041CG9997: p = 0.048	none detected	n/a
*vkg*	extracellular matrix component	female ST, FB, heart, TG, brain, head	CG17575: p = 0.007	none detected	n/a
***CG5630 (hdly)***	unknown	female ST, SG, crop, tubule, hindgut, midgut	CG17575: p = 0.005	near-complete	**KD: 15/27** **cont: 3/31** **p = 0.0002**

Expression based on data from FlyAtlas [64]. Predicted functions are from FlyBase electronic annotations. Bold indicates statistical significance for positive candidates. Abbreviations are as follows: AG = accessory gland; ST = spermatheca; FB = fat body; TG = thoracicoabdominal ganglion. For examples of near-complete and partial knockdown, see Figure S7. KD: knockdown, cont: control. The 4-day recepetivity assay column shows the number of females remating out of the total number of females tested for each condition; *p*-values are from Fisher's exact tests.

### ERC reveals new candidate SP network proteins

Since we detected positive evolutionary correlations between known SP network proteins, we applied the ERC method prospectively to identify new candidate network members. For this analysis, we calculated pairwise correlations using the reproductive protein data set described above, and we focused specifically on correlations between the known SP network proteins and the 434 proteins that comprised the sets of secreted Sfps and proteins present in the female reproductive tract. To identify candidates, we queried each of five network proteins (CG1652, CG1656, CG9997, CG17575 and SP) against the 434 Sfp and female proteins. SPR was not used as a query because it has additional ligands that do not appear to function in reproduction [62], [63]. Thus, SPR may need to maintain interactions with multiple proteins, which may dampen signals of correlated evolution with any single interacting partner. Seminase was excluded because unambiguous orthologs were found in only five species, which would cause low statistical power.

We found 111 proteins (55 Sfps, 56 female proteins) that showed a significant correlation (*p*<0.05) with at least one of the five network proteins. From this group, we selected 21 candidates for further testing, each of which showed a significant (*p*<0.05) level of ERC with multiple SP network proteins and/or a highly significant (*p*<0.01) level of ERC with at least one network protein (Table 1). We tested each candidate in Table 1 by using RNAi to knockdown expression of the gene in the appropriate sex; five of the 21 candidates showed no evidence of knockdown by RT-PCR and were excluded from further analysis. For the remaining 16 candidates, we screened for genes whose knockdown caused a significant increase in female remating receptivity four days after an initial mating.

Of the 16 candidates that were at least partially knocked down by RNAi, five showed highly significant effects on 4-day remating receptivity (Table 1). Knockdown of the remaining 11 candidates caused no significant increase in female receptivity. This latter result could be due in some cases to insufficient knockdown or to functional redundancy with other Sfps or female proteins. Alternatively, these proteins may not function in the SP network. Of the positive candidates, three genes (*CG14061*, *CG30488* and *CG12558*) are expressed specifically in the male accessory glands [64]; at least two of them (*CG14061* and *CG30488*) encode proteins that are transferred to females as Sfps at mating [33]. The other two positive candidates, *CG3239* and *CG5630*, are each expressed in the female's spermathecae, as well as in other non-reproductive locations [64]. *CG5630* is also expressed in the female's seminal receptacle [39].

### ERC signatures, but not genomic location, predicts an additional SP network protein

One striking feature of several of the new candidate network genes was their genomic positioning next to previously known SP network genes (Table S2). This pattern was previously observed for the SP network lectins, *CG1652* and *CG1656*, which are believed to have arisen from an ancient gene duplication event [33], [34]. We found that three additional pairs of network genes (*CG9997* and *CG14061*, *CG17575* and *CG30488*, and *CG3239* and *SPR*) are also located in tandem with one another. For two of these pairs, the tandemly-located genes encode proteins in the same biochemical category (*CG9997* and *CG14061* each encode predicted serine protease homologs, and *CG17575* and *CG30488* each encode predicted CRISPs), but in contrast to the situation with the lectins *CG1652* and *CG1656*, we do not find unambiguous evidence that either the protease or the CRISP cluster arose by tandem gene duplication. However, regardless of each cluster's origin, it is possible that such genomic clustering enables the co-regulation of genes that function in a common pathway [65].

In the *CG17575*/*CG30488* cluster, we found a third annotated gene that encodes a seminal fluid protein of the same predicted functional class as the other cluster members: *CG30486*, which encodes a predicted CRISP. Similarly, we observed a known Sfp gene encoding a predicted serine protease homolog, *CG34295*, immediately upstream of *CG12558*. While neither *CG30486* nor *CG34295* was identified by our ERC analysis, we hypothesized that their shared locations with known or candidate SP network members could indicate their involvement in the SP network. However, when each of these additional genes was knocked down individually, we observed no effect on female remating receptivity 4 days after mating (Table 2). Thus, either these neighboring genes are uninvolved in the SP network, or they function in the network in a completely redundant role. Alternatively, their degrees of knockdown may have been insufficient to produce a phenotype.

**Table 2:** Tests of neighboring genes and additional ERC candidates for 4-day receptivity phenotypes.

Gene Name	Predicted functional class	Expression pattern*	Significant ERC results	Amount of knockdown	4-Day Receptivity Assay
*CG30486*	CRISP	male AG	none (neighbor to *CG17575* and *antr*)	near-complete	KD: 0/29cont: 0/26p = 1.00
*CG34295*	serine protease homolog	male AG	none (neighbor to *intr*)	partial	KD: 0/24cont: 0/26p = 1.00
*sda*	alanine aminopeptidase	ubiquitous, including female ST	frma: p = 0.0051CG17575: p = 0.0185SP: p = 0.0262hdly: p = 0.0268	near-complete	KD: 5/29cont: 3/31p = 0.47
***Esp***	sulfate transporter	female ST, hindgut, brain, ovary, testes	antr: p = 0.0036CG9997: p = 0.0143	partial	**KD: 21/35** **cont: 2/34** **p<0.0001**

Abbreviations for expression patterns follow those listed in Table 1.

We also asked whether signatures of ERC between these new candidates and the rest of the large sets of seminal fluid or female proteins might identify additional network proteins (Figure S1C). To this end, we used RNAi to test two additional female genes that showed highly significant ERC levels with at least one new candidate protein (Table 2). One of these genes, *epidermal stripes and patches* (*Esp*), showed a highly significant effect on female remating receptivity. Taken together with the results above, these data suggest that ERC has strong sensitivity to detect new candidate members of the SP network.

### Additional RNAi lines confirm the SP network phenotypes

To confirm that the receptivity and fertility effects we observed in the above RNAi experiments were not due to RNAi off-target effects and/or insertions of RNAi-triggering constructs into essential genes, we first used UP-TORR [66] to analyze each line's RNAi-triggering sequence against all current *D. melanogaster* gene annotations. No off-target transcripts were predicted for any RNAi construct used. We then performed receptivity and long-term fertility assays (see Methods and below) on additional RNAi lines, where available, that controlled for either the site of the UAS-RNAi construct insertion (for *CG5630* and *Esp*) or both the insertion site and the hairpin sequence used to trigger RNAi (for *CG30488* and *CG3239*). (No additional RNAi lines exist for *CG14061* or *CG12558*). These tests (summarized in Table S3) confirmed the receptivity and fertility phenotypes seen with the first lines tested for *CG30488* and *Esp*. Likewise, knockdown of *CG5630* by a second hairpin showed a strong effect on fertility and a marginally significant effect on receptivity. Knockdown of *CG3239* by a second hairpin also replicated a strong effect on fertility, but showed no significant effect on receptivity. However, RT-PCR revealed that with this hairpin, *CG3239* transcript levels were only partially knocked down, which could explain the less severe phenotype. Because of the high degree of replication, results reported below come from experiments performed on the original lines (details of which are described in Table S1).

### ERC-identified candidates show additional receptivity and fertility phenotypes consistent with SP network function

To evaluate whether each of these six genes was required only for extended female non-receptivity, we next tested each positive candidate for effects on remating receptivity at 1 day after an initial mating. As shown in Table 3, in no case did knockdown of a candidate gene cause an increase in short-term receptivity. Thus, rather than having general effects on female post-mating behavior, each candidate is required specifically for the long-term loss of female receptivity to remating. This phenotype is consistent with a malfunction in the SP network [20], [21]. In females mated to SP network knockdown males, SP transferred at mating but not bound to sperm is sufficient for full fertility and non-receptivity 1 day after mating. However, if SP cannot bind to sperm, it is no longer detected in the reproductive tract by 4 days after mating [19].

**Table 3:** Tests of female remating receptivity 1 day after an initial mating.

Gene	Results	FET *p*-value
CG14061	KD: 3/26, cont: 1/28	0.34
CG30488	KD: 0/32, cont: 4/26	0.0352*
CG12558	KD: 0/14, cont: 2/15	0.48
CG3239	KD: 3/37, cont: 2/39	0.67
CG5630	KD: 1/21, cont: 1/28	1.00
Esp	KD: 3/23, cont: 0/23	0.23

Result not in the expected direction for non-functioning SP pathway.

KD: knockdown, cont: control, FET: Fisher's exact test.

**Figure 2:**
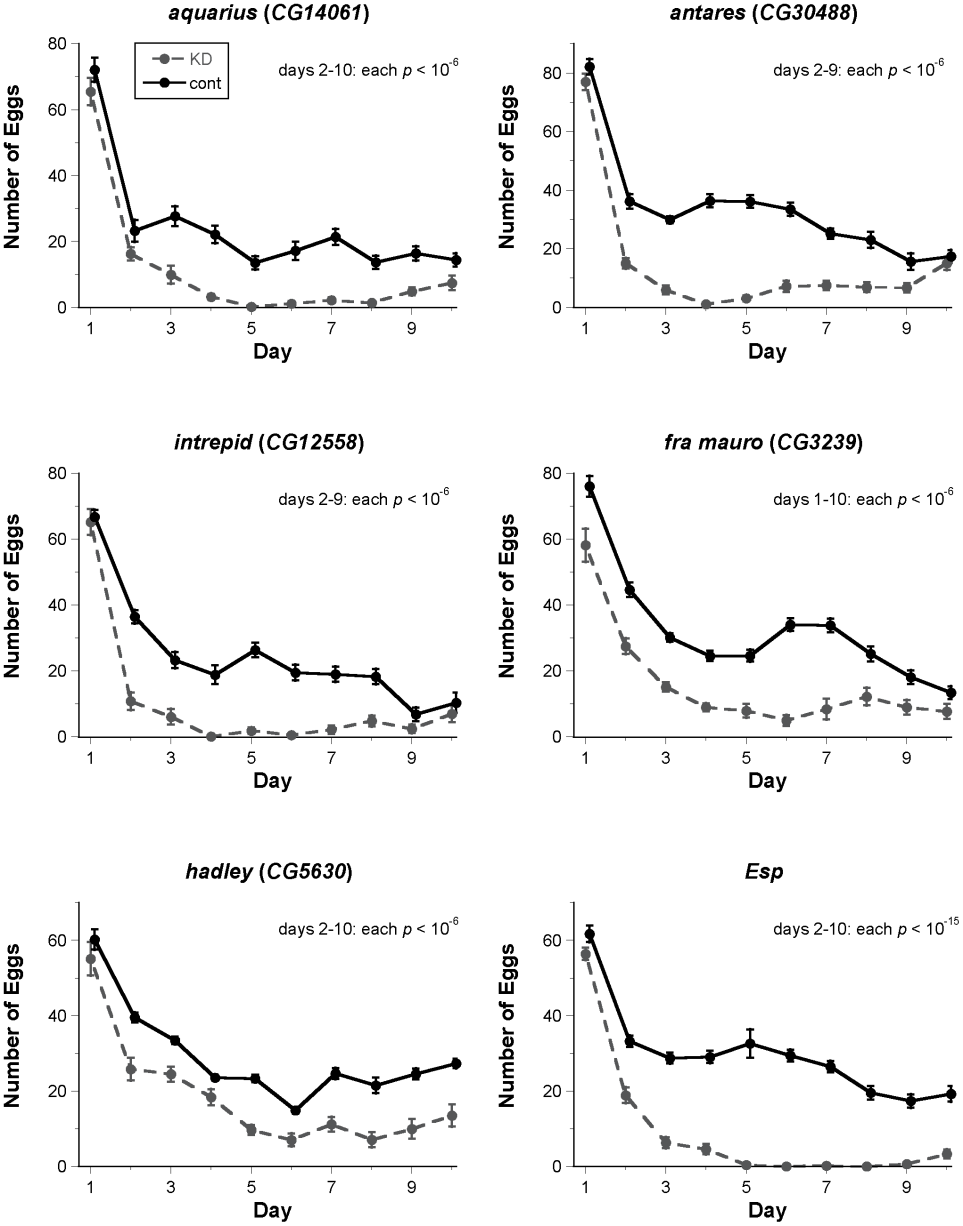
Fertility assays for new candidate SP network proteins identified by ERC. Each graph depicts the mean (± SE) number of eggs laid on each day of a 10-day fertility assay (knockdown: KD, dashed line; control: cont, solid line). For each male-expressed gene, knockdown or control males were mated to wild-type females. For each female-expressed gene, wild-type males were mated to knockdown or control females. Knockdown of each gene shown had a highly significant effect (corrected *p*<10^−6^ in all cases) on overall fertility; results of statistical testing for fertility on each day of the assay are shown on each graph. Control data points are offset horizontally from knockdown data points to facilitate comparison, but all flies in each experiment were transferred from one vial to the next at the same time each day. Samples sizes for each treatment range from 11 to 28. One representative biological replicate (out of 2–3 for each gene) is shown.

We reasoned that if these six positive candidates affect the function of the SP network, they should also affect long-term fertility, which requires the long-term storage and utilization of SP [17], [18], [20], [26], [28]. Consistent with a role in the SP network, each new protein was required for full fertility over the course of a 10-day assay (Figure 2). Males knocked down for *CG14061*, *CG30488* or *CG12558* induced normal levels of egg-laying and progeny production in females for the first day after mating, but these measures declined relative to controls as early as the second day after mating. Females knocked down for *CG5630* or *Esp* showed the same pattern of normal fertility on day 1 after mating, but reduced fertility in the following days. Females knocked down for *CG3239* had significantly reduced egg-laying and progeny production even on the first day after mating, mimicking the effects of knocking down *SPR* (Figure 2, Figure S2). These knockdown females then continued to have lower egg and progeny production throughout the assay. We further observed that knockdown of any male gene or of the female gene *Esp* had no significant effect on egg-hatchability, while knockdown of the remaining female genes caused hatchability to be significantly lower (Figures S3, S4). This effect was most pronounced in *CG3239* knockdown females, and much less severe in *CG5630* and *SPR* knockdown females. Effects on hatchability were unlikely to be due primarily to reduced viability of offspring inheriting both the UAS-RNAi construct and the GAL4 driver (see Text S1).

Thus, each of these six candidates identified by ERC is required for both the long-term loss of remating receptivity and the long-term maintenance of fertility. In our subsequent results and discussion, we adopt new names for the previously unnamed genes: male-expressed genes are named after lunar modules used in the Apollo space program (CG14061: *aquarius*; CG30488: *antares*; CG12558: *intrepid*), and female-expressed genes are named after sites on the moon at which Apollo missions landed (CG3239: *fra mauro*; CG5630: *hadley*).

The new male genes encode proteins predicted to belong to functional classes often found in insect and mammalian seminal fluid [33],[34],[67]–[69] and already represented in the SP network. Like CG9997, *aquarius* and *intrepid* encode serine protease homologs [70]; like CG17575, *antares* encodes a cysteine-rich secretory protein. In females, *fra mauro* encodes a protein that contains a partial, predicted neprilysin protease domain. Neprilysins are a class of protease that preferentially cleave prohormones and neuropeptides and are important for male and female fertility in mammals [71]–[73] and *Drosophila* (J. Sitnik et al. submitted). Neither annotated isoform of fra mauro is predicted by SignalP [74] to be secreted or extracellular, raising the question of how this protein could interact with SP network proteins. Inspection of the 5′ untranslated region of *fra mauro* revealed the presence of a potential alternative initiation codon, which is followed by a region predicted by SignalP to encode a functional secretion signal sequence. RT-PCR analysis on female cDNA found that a product could be amplified when a forward primer is placed in this region (data not shown), raising the possibility that an alternative isoform of the protein may be secreted and thus more accessible to other network proteins. In addition, we found this alternative start codon and secretion signal to be conserved in at least 11 of 12 *Drosophila* species analyzed (the *D. willistoni* genome sequence contains a sequencing gap in this region), which provides strong evidence that this secreted protein isoform is functionally important (Figure S5). The hadley protein is predicted to be secreted, but its potential functional class remains unknown, as neither conserved domain searching [75] nor three-dimensional structural modeling [76] could identify a conserved protein domain. The *Esp* gene was initially identified as a target of homeotic genes [77] but is, otherwise, poorly characterized. While the Esp protein is not predicted to be secreted, it shows homology to transmembrane sulfate transporters. In adults, *Esp* is expressed predominantly in the spermathecae [64], with additional expression reported in the seminal receptacle [39].

### Molecular characterization of new SP network proteins

**Figure 3:**
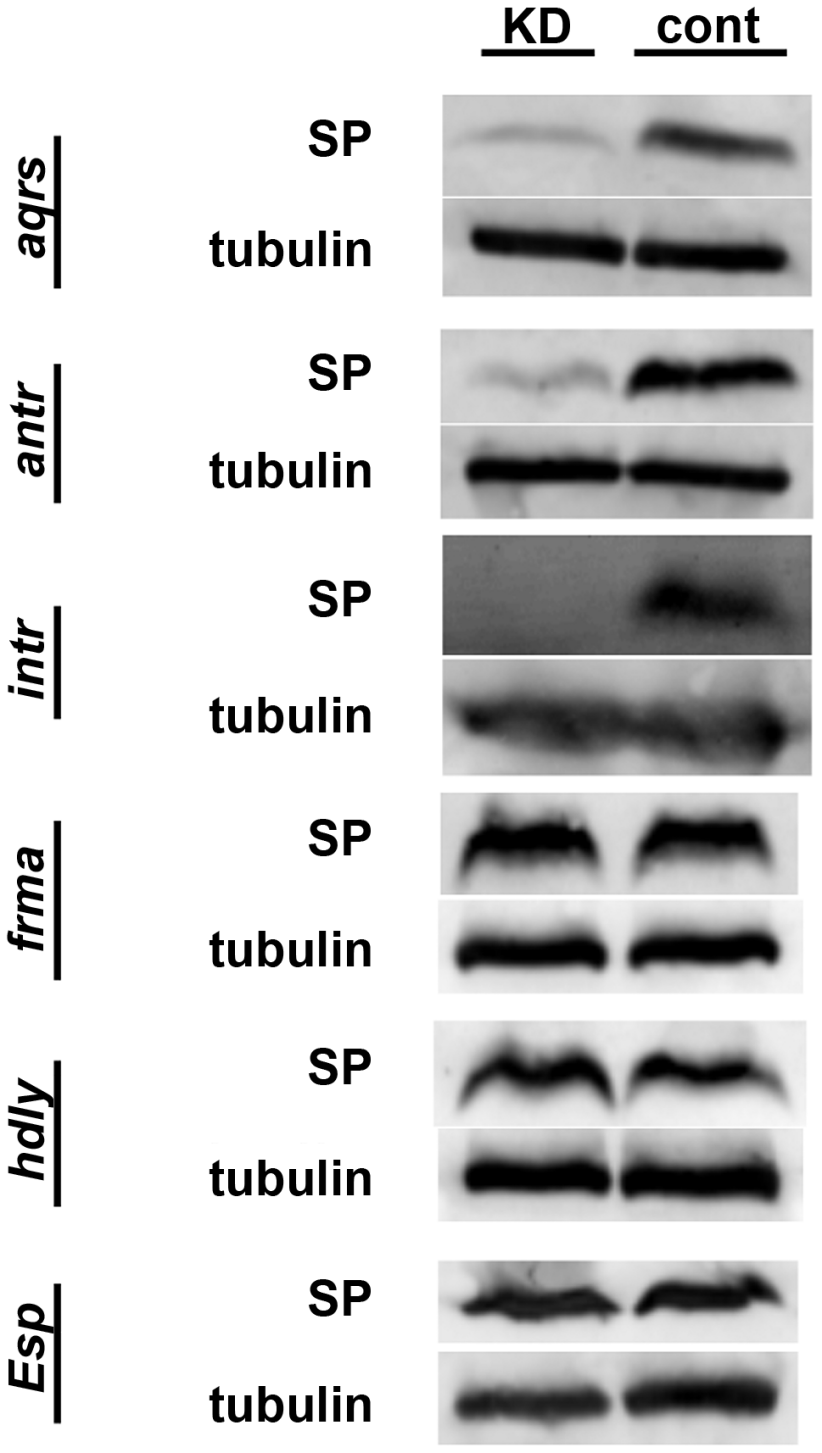
SP retention in mated females, 4 days after mating. Western blots probed with antibodies to SP or alpha-tubulin (loading control). Proteins were isolated from lower female reproductive tracts 4 days after mating. Gene names to the left of each pair of blots indicate which gene was (KD) or was not (cont) knocked down in the mating pair. Across all experiments, the number of female reproductive tract (RT) equivalents used for each condition ranged from 13 to 20; however, for any given gene, the number of RT equivalents compared between KD and control was within 2 RTs.

**Figure 4:**
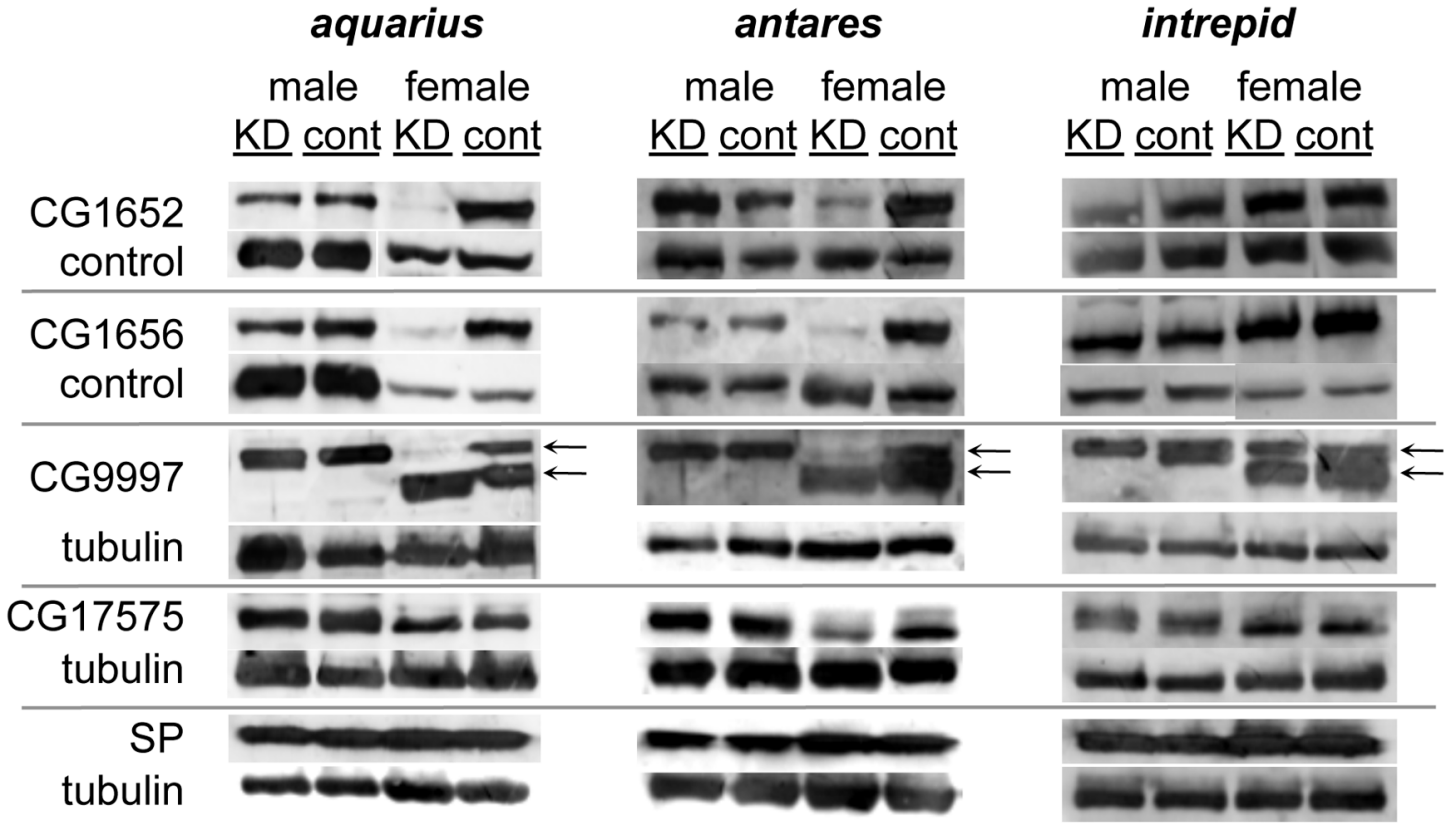
Production, transfer and processing of SP network proteins in males knocked down for *aquarius*, *antares* or *intrepid*. Western blots were probed with either an antibody to an SP network protein or a loading control. Alpha-tubulin was used as the loading control for blots of CG9997, CG17575 and SP. Since CG1652 and CG1656 sometimes co-migrated with tubuiln, loading controls for these proteins were either a consistently observed cross-reactive band or tubulin. Proteins were isolated from male reproductive tracts (“male” columns) or lower female reproductive tracts dissected 1 hour after the start of mating (“female” columns). “KD” indicates males knocked down for *aqrs*, *antr* or *intr* or females mated to a knockdown male, while “cont” indicates control males or females mated to a control male. Arrows next to the blots for CG9997 indicate the ∼45 (top) and ∼36-kDa (bottom) forms of the protein [21]. Within each blot, the amount of RT equivalents loaded for each sex was equal. Across blots, male lanes contain 0.5–1 RT equivalents; female lanes contain 2–4 RT equivalents.

We next sought to position these six new proteins in the SP network. To do so, we first used Western blotting to test whether SP was successfully stored over the long-term in mates of knockdown males or in knockdown females. In wild-type matings, SP is readily detectable from dissected female seminal receptacles (SRs) 4 days after a mating. However, knockdown of any of the known SP network proteins eliminates this retention [21], [28]. We observed that wild-type females mated to males knocked down for *aquarius*, *antares* or *intrepid* showed little or no SP at 4 days after mating (Figure 3). These reduced levels of SP were not due to less SP having been transferred at mating (see Figure 4). These results suggested that male proteins *aquarius*, *antares* and *intrepid* are each required for network function at a step upstream of SP binding sperm in the SR. By contrast, when wild-type males were mated to *fra mauro*, *hadley* or *Esp* knockdown females, normal levels of SP were observed at 4 days after mating (Figure 3). Thus, these female proteins may be necessary for the utilization of SP after it becomes stored in the SR or may be required for proper SP-SPR signaling.

To further determine where the new male proteins fit into the network, we examined the production of the known SP network proteins in males knocked down for *aquarius*, *antares* or *intrepid* (Figure 4). In all cases, we observed no difference in the production of SP, CG1652, CG1656, CG9997 and CG17575 between knockdown and control males (Figure 4; compare lanes for knockdown and control males). We then tested whether knockdown males could transfer these proteins to females and examined their processing in female reproductive tracts. Males knocked down for *intrepid* transferred all proteins at equivalent levels to controls, and females mated to these males showed normal CG9997 processing [21] in their reproductive tracts. Males knocked down for *aquarius* or *antares* transferred normal levels of SP, CG9997 and CG17575, but much lower levels of CG1652 and CG1656 (Figure 4; compare lanes for females mated to *aquarius* or *antares* knockdown or control males). Consistent with the absence of these proteins in females after mating [21], the post-mating processing of CG9997 was also disrupted, with mates of knockdown males showing an increased level of the 36-kDa form of CG9997 relative to the 45-kDa form of this protein. We also examined the production and transfer of seminase and observed no differences between knockdown and control flies for each gene (data not shown).

**Figure 5:**
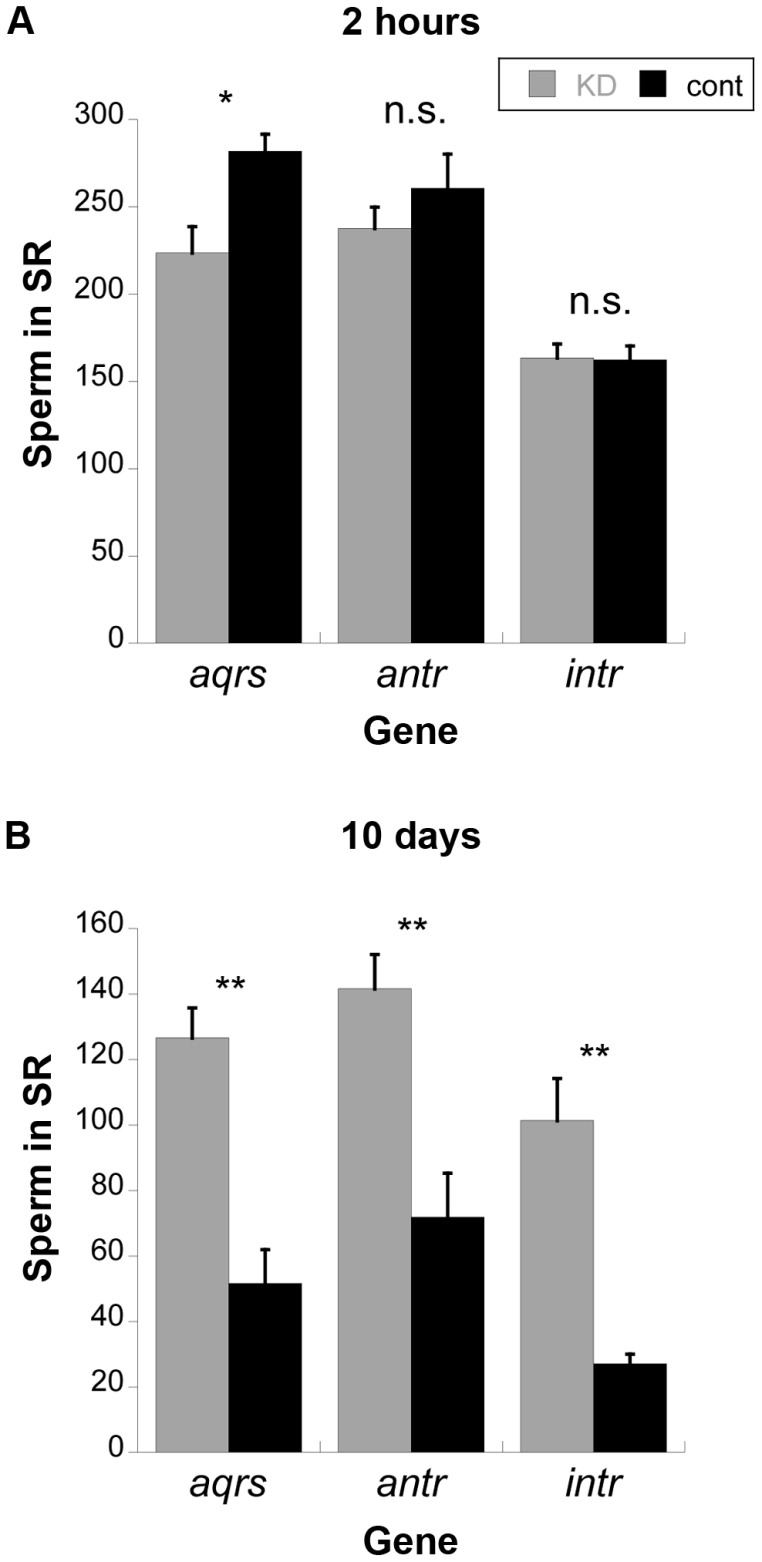
Average number of sperm stored in the seminal receptacles (SR) of wild-type females mated to knockdown or control males for new SP network proteins. Average number of sperm in female SRs at 2(A) or 10 days (B) after mating to *aqrs*, *antr* or *intr* knockdown (KD, gray) or control (cont, black) males. Each bar indicates the mean; error bars indicate 1 standard error. *, *p*<0.01; **, *p*<0.002; n.s. = not significant. Samples sizes for each treatment range from 11 to 18.

Because SP is required for the release of sperm from storage [27], we examined sperm storage and retention in the SRs of females mated to males knocked down for each of these genes (Figure 5). At 2 hours after mating, sperm from *antares* and *intrepid* males were present in the SR at equivalent levels to controls, while sperm from *aquarius* males were present at slightly lower levels. However, by 10 days after mating, mates of control males had largely depleted their stores of sperm in the SR, while mates of males knocked down for any of the three genes showed significantly higher numbers of sperm. Taken together with the lack of SP retention (see Figure 3), these data confirm that male proteins *aquarius*, *antares* and *intrepid* are each required for SP to become bound to sperm. Disruption of this binding, in turn, inhibits the ability of sperm to be released from the seminal receptacle. This inability to release sperm from storage likely contributes to the reduction in long-term fertility when each of these male genes is knocked down (Figure 2).

**Figure 6:**
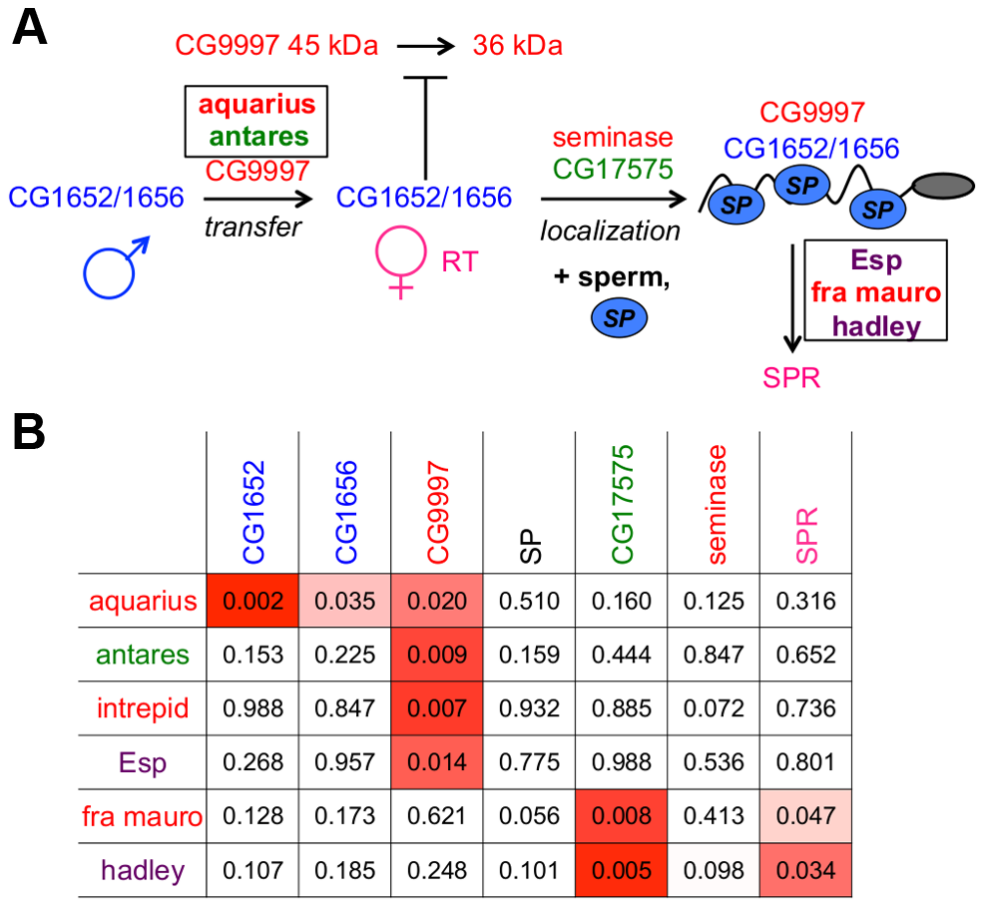
An expanded network of proteins is required for SP to bind sperm and to be utilized in mated females. (A) The SP network. Colors of protein names indicate predicted protein functional classes: red = protease or protease homolog; green = cysteine-rich secretory protein (CRISP); dark blue = C-type lectin; light blue oval = SP; purple = unknown function. Boxes indicate proteins discovered by ERC; other proteins were described previously [21], [28]. Intrepid acts upstream of SP-SPR signaling, but at present we cannot position it further. (B) New members of the SP network function at steps consistent with their signals of ERC. New network proteins are shown in rows; known network proteins are shown in columns. Each cell indicates the empirical *p*-value associated with the protein's pair ERC value. *P*-values less than 0.05 are shaded in red; more intense shading indicates a stronger correlation.

Taken together, our results allow us to place aquarius, antares, fra mauro, hadley and Esp into the SP network (Figure 6A). The male proteins aquarius and antares act at the same step of the network as CG9997, as each of these proteins is required for the transfer of CG1652 and CG1656. The female proteins fra mauro, hadley and Esp appear to act at the downstream end of the network, after SP has bound to sperm. At present, we are unable to position intrepid within the network, though its effect on SP retention (Figure 3) suggests that it acts upstream of SP-SPR signaling.

### A protein's evolutionary correlations reflect its position in the SP network

When comparing the positioning of these six new proteins in the network to their patterns of ERC with the previous known seven network proteins (Figure 6B), we observed that the new male proteins showed their strongest correlations with the upstream players of the network. In particular, each new male protein showed a significant correlation with CG9997, which functions in the same step of the network (CG1652/CG1656 transfer) as aquarius and antares. At the downstream end of the pathway, two of the new female proteins showed their strongest correlations with downstream players in the network, including SPR, which is consistent with their potential functions. Thus, the patterns of ERC observed between new and established network proteins are consistent with the steps in the network in which these new proteins are found to act.

## Discussion

We have used signatures of covariation in protein evolutionary rates to investigate interactions between proteins that are required to maintain post-mating responses in *Drosophila* females. We first found that, as a group, proteins known to act in the SP network [20], [21], [26], [28] showed a significant signature of ERC. We then used ERC to screen 434 male Sfps and female reproductive tract proteins for those that correlated strongly with members of the SP network. RNAi functional testing of 16 top candidates identified five proteins that are each required for long-lasting SP responses in females, including reducing a female's willingness to remate and boosting female egg production. Additional tests of two candidates that showed high ERC with these new genes revealed a sixth network protein. The new male proteins, *aquarius*, *antares* and *intrepid*, act in the upstream part of the network: loss of any one of these proteins prevents SP from becoming bound to sperm, which in turn prevents sperm from being released from storage. Because SP binds to sperm in females knocked down for *fra mauro*, *hadley* or *Esp*, these proteins may affect the ability of SP to be used in females and/or may be required for normal SP-SPR signaling. Interestingly, the strongest evolutionary correlations between these new proteins and the known members of the network often occurred between pairs of proteins that appear to act in the same part of the pathway. These results verify the utility of ERC and suggest that this metric may be used prospectively to identify candidates acting in a particular part of a pathway.

### ERC efficiently identifies new types of network proteins

Our results suggest that ERC successfully prioritized a large set of proteins for detailed functional testing; the observed success rate was six positive hits out of 18 candidates tested, and this rate could be higher if genetic redundancies or insufficient knockdown prevented positive results for some candidates. This rate likely represents a significant enrichment of network genes because if the same success rate were applied to the full list of 434 reproductive proteins, it would imply that there are 145 long-term mating response genes waiting to be discovered in that list alone. Although this is a formal possibility, this number seems high. Importantly, ERC allowed us to explore new functional classes of protein from the female reproductive tract. Previous studies [20], [28] chose male-expressed candidates based on molecular classes that were known to function in sperm storage and fertilization. In contrast, ERC directed us to proteins that unlikely would have been selected for screening, as fra mauro was not annotated to be extracellular and hadley had no predicted functional class. We can also prescribe a strategy to improve ERC analysis by retrospectively analyzing the positive candidates. Very strong correlations (*p*<0.01) tested positive more often, so future applications of this method could focus on single, strong correlations rather than those proteins that correlate more weakly (*p*<0.05) with multiple network members. Finally, we note that several reproductive proteins showed strong signals of ERC with the SP network but were not quickly testable because RNAi lines were not available. In cases like these, emerging technologies such as the CRISPR/Cas9 system that is now being optimized for *Drosophila* [78], [79] may in the future enable null mutants to be generated, which could potentially expand the SP network further.

### Possible functions for new network proteins

By expanding the SP network to include new proteins from both sexes, our results provide a more complete picture of how SP controls female post-mating responses. Until now, SPR was the only known female regulator of SP action [26], but our results show that fra mauro, hadley and Esp are also necessary for sperm-bound SP to exert its long-term effects on females. In addition to their expression in the spermathecae, each of these female genes is expressed in regions other than the female reproductive tract [64]. SPR follows the same pattern: it is expressed in several reproductive regions [26], including the spermathecae, and elsewhere in the adult female. However, only six SPR-expressing neurons in the reproductive tract are required for the SP response [23]–[25]. It is also interesting to compare the fertility phenotypes for *fra mauro*, *hadley*, *Esp* and *SPR* knockdown females (Figure 2). Knockdown of *fra mauro* or *SPR* causes both a long-term fertility deficit and an immediate reduction in egg-laying in the first 24 hours after mating. In contrast, *hadley* or *Esp* knockdown females show normal fertility on day 1, but then have reduced fertility over the following days. Assuming that the extent of gene knockdown was sufficient to reveal null-like phenotypes, one possible model to explain these results could be that fra mauro is necessary to facilitate SP-SPR signaling, while hadley and Esp are necessary for the efficient release of SP from stored sperm. SP-SPR signaling is required for full fertility at all time points after mating (Figure S2 and [26]), but impaired release of SP from sperm affects fertility only after day 1 [19]. Another possibility is that fra mauro is required to coordinate temporally the release of sperm from storage when eggs are ovulated and ready to be fertilized. Furthermore, while knockdown of *fra mauro*, *hadley* and *SPR* each caused a reduction in egg hatchability, the magnitude of this effect was by far the greatest for *fra mauro* (Figures S3, S4). Thus, in addition to laying significantly fewer eggs than controls (Figure 2), *fra mauro* females also experience far lower egg-to-adult viability. Finally, it is interesting to observe that Esp is a predicted sulfate transporter. In mammalian systems, anion concentration in the female reproductive tract is critical for proper sperm function and fertility [80]. In *Drosophila*, it is possible that attenuation of extracellular levels of anions such as sulfate in the sperm storage organs affects Sfp-sperm binding, sperm storage, SP release, or another process required for SP network function.

Two observations suggest that interactions between SP network proteins may begin in the male. First, *CG9997*, *aquarius* and *antares* are each required for lectins CG1652 and CG1656 to be transferred efficiently to females [21] (Figure 4). It is possible that one or more of the former proteins may bind to either lectin protein as Sfps transit the male reproductive tract during mating. Such binding could protect the lectins from proteolysis or modification. For instance, *CG9997* and *aquarius* both encode serine protease homologs that are predicted to have inactivating mutations in their active sites [70]. It has been speculated that such inactive proteases could act as competitive inhibitors of proteolytic processing by binding to processing targets, rendering them less accessible to the numerous active protease in the seminal fluid [81]. Second, it is presently unclear whether intrepid is transferred at mating, as previous proteomic experiments have not detected this protein in mated females [33]. While intrepid may be transferred but poorly detectable in mated females (e.g., due to low abundance or rapid degradation), it may, alternatively, act in males to modify or activate another network protein(s). Processing of Sfps within males is observed in other cases. For example, the *Drosophila* seminal metalloprotease CG11864 is processed in the male reproductive tract during transfer to females, and this processing is required for CG11864 to mediate the processing of additional Sfps in the female reproductive tract [28], [82] (B. LaFlamme, F. Avila et al., submitted). In nematodes, interactions between a protease, TRY-5, and a protease inhibitor, SWM-1, regulate the activation of sperm during transit through the male reproductive tract [83]–[85]. Thus, it will be interesting to determine whether any members of the SP network are the agents or targets of processing within the male reproductive tract. If network proteins are modified while still in the male, this process may be regulated spatially and temporally by the sequestration of interacting components into distinct compartments of the reproductive tract, including the ejaculatory bulb [86] and vesicles found in secondary cells of the accessory gland [87], [88]. Such compartmentalization could ensure that interacting proteins do not encounter each other until the appropriate time during or after mating.

### Evolution of the SP network

Our results, combined with previous work [20], [21], [26], [89], suggest that at least 13 proteins participate in the SP-mediated post-mating response in female *Drosophila melanogaster*. How did this complex network arise, and how have its members evolved? Orthologs of the sex peptide receptor (SPR) are found in diverse insect taxa, including mosquitoes, silkworms and moths, and these receptors are responsive to stimulation by *D. melanogaster* SP [26], [90]. However, SP has not been identified outside of Diptera; a putative SP ortholog was identified by bioinformatics in *Anopheles* [91], but the short length of SP makes it difficult to detect orthologs in other species, including some drosophilids. Furthermore, the female post-mating responses of insects with SPR orthologs often differ substantially from those of the *melanogaster* group of *Drosophila*. For example, *D. mojavensis* females re-mate more readily than *D. melanogaster* females [92], and while *A. gambiae* females become unreceptive to further courtship after a single mating, this behavioral change does not require the transfer of sperm [93].

Within the genus *Drosophila*, other members of the network show different levels of evolutionary conservation. We identified orthologs of CG1652, CG1656, CG9997 and CG17575 in 11 of 12 sequenced *Drosophila* species (all but the most distant species, *D. grimshawi*). Most of the new proteins we identified share this broad distribution throughout the genus. Hadley and fra mauro are found in all 12 species, but appear not to have orthologs in sequenced mosquito species (data not shown). Aquarius and antares show the same species distribution as CG1652, CG1656, CG9997 and CG17575. Esp orthologs are found in only ten species, but these include one member of the more distantly related Drosophila clade, *D. mojavensis*, suggesting an older origin for this protein. In contrast, intrepid and seminase appear to have evolved more recently, with orthologs detectable only in the Sophophora clade. Orthologs of intrepid were found in 9 of 12 species (all but *D. virilis*, *mojavensis* and *grimshawi*), while seminase orthologs were detected only in *D. melanogaster*-*D. ananassae*. Taken together, these varying degrees of evolutionary conservation suggest that the SP network, as it presently functions in *D. melanogaster*, may have evolved in pieces over time. Indeed, the emergence of the full SP network correlates with changes in remating rate. Frequent mating (daily or more than once per day) was inferred to be the ancestral condition for drosophilids, while less frequent mating is derived and appears in those species (*D. melanogaster* through *D. pseudoobscura*) that have all or nearly all of the SP pathway components [94].

Some reproductive proteins of many species have evolved under positive selection [95]–[97]. One proposed explanation for this pattern suggests that males and females may experience sexual conflict over some aspect of reproduction (e.g., the rate of female remating). Substantial evidence suggests that sexual conflict occurs in *D. melanogaster* [98]–[100] and is mediated by SP [101]. At the molecular level, the result of sexual conflict could be continual coevolution between male and female protein sequences. Population genetic studies have detected evidence of recent selective sweeps on SP [102] and CG9997 [103], but most other members of the network appear well conserved [33]. One possible explanation centers on the observation that SPR is sensitive to multiple ligands [26], [62], [63], which may constrain its ability to coevolve with SP and thus reduce the requirement for constant coevolution. It will also be instructive to examine the molecular evolution of all network members across the *Drosophila* phylogeny and to determine whether any have experienced bursts of positive selection on the same phylogenetic lineages, as might be predicted for proteins showing patterns of ERC [50].

### Conclusions

We have shown that signatures of evolutionary rate covariation can be used prospectively to identify new members of a protein network. In the context of the *Drosophila* SP pathway, this genomic approach allowed us to efficiently screen hundreds of known reproductive proteins so as to prioritize candidates for functional analysis, thereby identifying new long-term mating response proteins from both males and females. Interestingly, male and female proteins appear to participate in distinct sections of the SP network, and this separation was reflected in their signatures of correlated evolution. We believe that the ERC approach will be broadly applicable to identifying new members of other protein networks in any taxa for which comparative genomic data are available.

## Methods

### Reproductive proteins data sets

We used a combination of published proteomic and transcriptomic data sets and genome-wide expression data to create three sets of reproductive genes used in the analysis: seminal fluid proteins (Sfps), female reproductive tract proteins, and sperm proteins. The first set consisted of 208 genes encoding Sfps that had been identified by mass spectrometry in the reproductive tracts of mated females [32], [33] or predicted secreted proteins from the male accessory gland [34]. The second set included 226 genes expressed in the female sperm storage organs. This set included the *D. melanogaster* orthologs of EST sequences identified from the spermathecae of *D. simulans* [36], [38] and EST sequences identified from the seminal receptacle of *D. melanogaster* [39]. We removed from these sets annotated housekeeping genes (e.g., ribosomal and mitochondrial proteins) since they were unlikely to interact with proteins in the SP network. Because EST sequencing may not sample all relevant genes, we then supplemented these genes with genes identified in FlyAtlas [64] to be predominantly expressed in the spermathecae (the only female sperm storage organ for which genome-wide expression data are available). The third set included 322 genes that encode proteins in the *D. melanogaster* sperm proteome [30], [31] and that were found in FlyAtlas to be predominantly expressed in the testis. This filtering was performed to enrich for proteins likely to function specifically in reproduction, since proteins involved in additional biological processes may interact with several partners and thus show dampened signals of ERC. While we used all three sets of genes (756 genes in total) for optimizing the ERC method (see below), we focused our further functional tests on ERC candidates identified from the seminal fluid and sperm storage organ gene sets (434 in total).

### Alignment of orthologous protein coding sequences from 12 species

We identified orthologous genes from 12 *Drosophila* species using a combination of high-throughput and manual searching. Protein amino acid sequences were produced by the *Drosophila* 12 Genomes project and downloaded from FlyBase (http://flybase.org) [61]. The species were: *Drosophila melanogaster*, *sechellia*, *simulans*, *yakuba*, *erecta*, *ananassae*, *pseudoobscura*, *persimilis*, *willistoni*, *grimshawi*, *virilis*, and *mojavensis*. Orthologs were identified using InParanoid, and the resulting groups were aligned by MUSCLE [104], [105]. Many alignments were missing species either due to evolutionary loss or missed gene annotation. To increase the number of species and thereby improve our power, we manually searched for unannotated genes in the 11 non-*melanogaster* species using a combination of tBLASTn and BLAT. This effort added 81 previously unannotated sequences to a total of 31 alignments.

### Genome-wide Evolutionary Rate Covariation (ERC) analysis across 12 *Drosophila* species

To perform ERC analysis, we first calculated the amount of amino acid divergence for each branch in the species tree for each of the 11,100 orthologous protein alignments produced above; this was done using ‘aaml’ of the PAML package [106]. Next, raw branch lengths were transformed into rates of evolution relative to the expected branch length. This projection operation, introduced by Sato et al. [58], removes the inherent correlation of all proteins due to the underlying species tree and improves the power of ERC to resolve functionally related protein pairs from unrelated pairs [55], [58]. Finally, we used these corrected branch-specific rates to calculate the correlations for all pairs of proteins, resulting in a proteome-by-proteome matrix of correlation coefficients, termed the ERC matrix. To limit the effect of outlier points, we limited all rates to 2 standard deviations from the mean.

In spite of our efforts (above) to improve species coverage, most alignments were missing at least one species. We set a minimum species threshold at 5, so species representation ranged from 5 to 12. This heterogeneity required us to create a flexible system to compare ERC results between different sets of species. A table of relative rates (projection operation, above) was produced for each unique set of species shared between protein pairs, resulting in 1,815 projections. Importantly, the distribution of ERC values varied depending on the particular set of species employed. For example, the variance of ERC values is consistently larger for smaller numbers of species (Figure S6). To correct for these effects we converted every observed ERC value in to an empirical *p*-value based on the observed distribution of ERC values for that particular set of species. The comparison of *p*-values allowed us to compare ERC results across all protein pairs. Hence, we report all ERC results as *p*-values ranging from 0 to 1, where a lower value indicates stronger evidence for rate correlation.

Significance testing for elevated ERC values in a set of proteins was performed using a proteome-wide permutation test (Figure S1A). The mean ERC value observed between all pairs in the tested set, such as the SP network, was compared to the mean ERC values of 10,000 sets of the same number of proteins randomly chosen from the entire proteome. A *p*-value for the tested set was computed as the proportion of random sets that had a mean ERC value equal to or greater than the tested set of proteins. Randomly chosen ERC values were taken from the same species-matched projections as in the observed set, which controlled for variation in ERC distributions due to different sets of species present in those genes.

The “reproductive protein only” analysis (Figure S1B–C) was performed as above, except that analysis was limited to the 756 Sfps, female proteins, and sperm proteins described above. We further limited this set to the 664 proteins that had detectable orthologs in at least 5 species. Significance testing for single pairs and for sets of proteins was performed as above, through empirical *p*-values. Calculations of pairwise correlations between pairs of known network proteins and between known network proteins and members of the sets of Sfps and female proteins were performed using this reproductive protein set.

### RNA interference (RNAi)

To knock down expression of candidate genes, we used a variety of RNAi lines and drivers. Most lines were second-generation (KK) RNAi lines provided by the Vienna *Drosophila* RNAi Center (www.vdrc.at) [107]; several others were either provided by the Transgenic RNAi Project (TRiP; Harvard University) [108] or constructed in house using the pVALIUM20 vector [109], [110] provided by the TRiP. When possible, we used the *tubulin*-GAL4 driver to knockdown genes ubiquitously, but in some cases knockdown with this driver caused lethality. When ubiquitous knockdown of a male-expressed Sfp gene caused lethality, we first attempted to use the *prd*-GAL4 driver [111] to knockdown expression in the accessory glands. However, we observed phenotypes consistent with SP network malfunction when this driver was crossed to a control background strain that does not induce RNAi. Thus, we instead used the *ovulin*-GAL4 driver [17] to knock down male Sfp genes. To knockdown female genes expressed in the spermathecae, we used the *Send1*-GAL4 driver [112], sometimes in combination with a UAS-Dicer2 sequence to enhance RNA interference. The RNAi line numbers, specific crosses and genetic controls used are given in Tables S1 and S3. All flies were reared on a 12 hr/12 hr light-dark cycle. Most crosses were performed at room temperature (22°C±1°); some were instead performed at 25° to attempt to induce greater knockdown.

We determined the degree of knockdown by using RT-PCR [20], [28] to measure the expression level of each RNAi-targeted gene in knockdown flies and their respective controls, using amplification of the *RpL32* transcript as a positive control (see Protocol S1 for further details). For *tubulin*-GAL4 knockdown, we analyzed RNA isolated from whole flies; for tissue-specific knockdown, we analyzed RNA isolated from dissected reproductive tracts. We qualitatively scored the degree of knockdown as “complete/near complete,” “partial,” or “no detectable knockdown”, and we chose for functional analyses only those genes (16 of 21 tested) that showed at least partial knockdown. Figure S7 shows knockdown levels for all positive candidates.

### Screens for reproductive phenotypes

For several days after an initial mating, females are reluctant to remate in a one-hour, single-pair test, but only if the SP network is functioning properly [19], [20]. Thus, we initially screened each candidate gene for its effects on a female's willingness to remate within 1 hour, 4 days after an initial mating, using previously described methods [20]. Positive candidates were then evaluated by the same assay for remating receptivity at 1 day after mating, and for fertility, fecundity and egg hatchability over 10 days after an initial mating. These assays were performed according to previously described methods, with minor modifications. For more detail, see Protocol S1.

### Confirmation of RNAi phenotypes

While all RNAi lines used above were designed to specifically minimize off-target effects [107], [108], we also confirmed that the phenotypes we observed were due specifically to the knockdown of the intended target. We first confirmed that all RNAi-triggering constructs had no predicted off-target effects against the most current *D. melanogaster* gene annotations [66]. We then tested an additional RNAi line for all genes for which such a line was available (*antares*, *fra mauro*, *hadley* and *Esp*). These tests controlled for either the insertion site of the RNAi-triggering construct or both the insertion site and the sequence of the RNAi-triggering construct, depending on which type of additional line was available. Details of these lines are given in Table S3. Finally, we note that our rate of positive hits in our screen (33 percent; 6 out of 18 ERC-identified candidates) is dramatically higher than previous estimates of RNAi effects on cell viability (maximum rate: 2.2 percent, including both true positive effects and potential off-targets) [113]. Thus, our results are unlikely to be due to off-target effects or general effects on cell viability.

### Western blotting

To examine the production, transfer and processing of known SP network proteins in flies knocked down for a newly identified candidate, we performed Western blot experiments using available antibodies to SP, CG1652, CG1656, CG9997 and CG17575 as previously described [21]. For each positive candidate, we first tested whether SP was retained on sperm over the long term by dissecting 13–20 lower female reproductive tracts for each treatment at 4 days after the start of mating (ASM). While the number of female reproductive tracts per lane across experiments varied within this range, pairs of samples being compared never differed by more than 2 tracts. Extracted proteins were run on 15% acrylamide gels, transferred to membranes, and then probed for SP and alpha-tubulin (as a loading control) as previously described.

For candidates that caused a reduction of SP levels in females at 4 days ASM, we then evaluated the production, processing and transfer of the known network proteins by testing for their presence in male reproductive tracts and in mated females at 1 hr ASM. Proteins were separated on 10.6% acrylamide gels and then transferred and probed for as described previously. Approximately 0.5–1 male reproductive tract equivalents and 2–4 lower female reproductive tract equivalents were loaded in each lane. While the number of female reproductive tract equivalents per lane varied between blots for different SP network proteins, comparisons between knockdown and control flies for any given protein were performed with an equal number of reproductive tracts in each lane. As a loading control for each blot, we primarily used alpha-tubulin. In cases where CG1652 and CG1656 co-migrated with alpha-tubulin, we also examined a consistently observed cross-reactive band.

## Supporting Information

Figure S1 Flow chart of ERC comparisons and screening. This diagram provides a conceptual view of the evolutionary rate covariation calculations described in the main text. (A) We first tested for whether the seven previously known members of the SP network showed a significant increase in their mean pairwise correlations by comparing them to sets of proteins drawn from the whole *D. melanogaster* proteome (*left side*). However, we also observed a slight but significant increased mean pairwise correlation when screening entire sets of reproductive proteins against the whole proteome (*right side*). (B) Because of this, we next controlled for the reproductive protein background effect by comparing the SP network proteins to randomly drawn sets of other reproductive proteins. The SP network proteins' mean pairwise correlation remained highly significant. (C) Finally, we screened five known SP network proteins against sets of male seminal proteins (Sfps) and female sperm storage organ proteins, 434 in total. We initially tested 16 highly correlated candidates and identified five new network members. We then screened these five new members against the large sets of reproductive proteins and identified a sixth new network member, Esp.
(PDF)

Figure S2 Fertility assay for females knocked down for *SPR*. This graph depicts the mean (± SE) number of eggs laid on each day of a 10-day fertility assay involving females knocked down for *SPR* (KD, dashed line; *n* = 16) and their controls (cont, solid line; *n* = 23). As previously reported, we observed a significant effect of knockdown on overall fertility (*p*<10^−6^), as well as significant differences on days 1–8 of the assay. Control data points are offset horizontally from knockdown data points to facilitate comparison, but all flies in each experiment were transferred from one vial to the next at the same time each day. These data are from one representative biological replicate.
(PDF)

Figure S3 Overall rates of egg hatchability during 10-day fertility experiments. Each boxplot shows the distribution of egg hatchability rates for matings involving knockdown (KD) or control (cont) flies for each candidate gene. The thick black line represents the median rate of egg hatching across the entire 10-day assay; thin lines indicate the first and third quartiles; dots indicate outliers that lie further beyond the edge of box than 1.5× the interquartile range. *P*-values below each graph indicate results from statistical testing; after Bonferroni correction, *p*<0.0083 are considered significant. These data come from the experiments depicted in Figure 2.
(PDF)

Figure S4 Day-by-day hatchability for female genes *fra mauro* (*CG3239*), *hadley* (*CG5630*) and *SPR*. Each point represents the total proportion of all eggs laid by all knockdown (KD) or control (cont) females that hatched on a given day during a 10-day fertility assay. These data come from the experiments depicted in Figure 2.
(PDF)

Figure S5 Alignment of protein sequences obtained by translating the 5′ untranslated region and the annotated coding region of the *fra mauro* gene in 12 *Drosophila* species.
(PDF)

Figure S6 ERC values are more tightly distributed when more species are available for analysis. For each graph, 10,000 pairs of proteins were chosen randomly from the entire *D. melanogaster* proteome. ERC values were calculated using protein sequences from either (A) five closely related species (*D. melanogaster*, *simulans*, *sechellia*, *yakuba* and *erecta*) or (B) all 12 fully sequenced species of *Drosophila* [61].
(TIFF)

Figure S7 RT-PCR results verify RNAi knockdown for positive ERC candidate genes. Each gel shows PCR amplicons from reactions performed with a template of: cDNA synthesized from either knockdown (KD) or control (cont) flies of the appropriate sex, *D. melanogaster* genomic DNA (gDNA), or, as a negative control, water (H_2_O). In all cases, flies knocked down for a candidate gene showed either complete/near-complete (*aqrs*, *antr*, *intr*, *hdly*) or partial (*frma*) knockdown. When possible, RT-PCR primers were designed so that multiple exons would be amplified, resulting in larger products when gDNA was used as a template.
(PDF)

Protocol S1 Supporting methods.
(PDF)

Table S1 RNAi lines, drivers and crosses used in this study.
(PDF)

Table S2 Genomic locations of SP network proteins in *Drosophila melanogaster*.
(PDF)

Table S3 Measures of receptivity and fertility for additional RNAi lines for positive ERC candidate genes.
(PDF)

Text S1 Supporting results text testing for whether the egg hatching defects observed in *fra mauro* or *hadley* females could be explained by reduced offspring viability.
(PDF)
