# Supplementary material for: Evolutionary Rate Covariation Identifies New Members of a Protein Network Required for Drosophila melanogaster Female Post-Mating Responses
Source: PLoS Genet. 2014 Jan 16;10(1):e1004108. doi: 10.1371/journal.pgen.1004108 (PMC3894160; doi:10.1371/journal.pgen.1004108)
Supplement: Figure S1 — Flow chart of ERC comparisons and screening. This diagram provides a conceptual view of the evolutionary rate covariation calculations described in the main text. (A) We first tested for whether the seven previously known members of the SP network showed a significant increase in their mean pairwise correlations by comparing them to sets of proteins drawn from the whole D. melanogaster proteome (left side). However, we also observed a slight but significant increased mean pairwise correlation when screening entire sets of reproductive proteins against the whole proteome (right side). (B) Because of this, we next controlled for the reproductive protein background effect by comparing the SP network proteins to randomly drawn sets of other reproductive proteins. The SP network proteins' mean pairwise correlation remained highly significant. (C) Finally, we screened five known SP network proteins against sets of male seminal proteins (Sfps) and female sperm storage organ proteins, 434 in total. We initially tested 16 highly correlated candidates and identified five new network members. We then screened these five new members against the large sets of reproductive proteins and identified a sixth new network member, Esp. (PDF) [file pgen.1004108.s001.pdf]

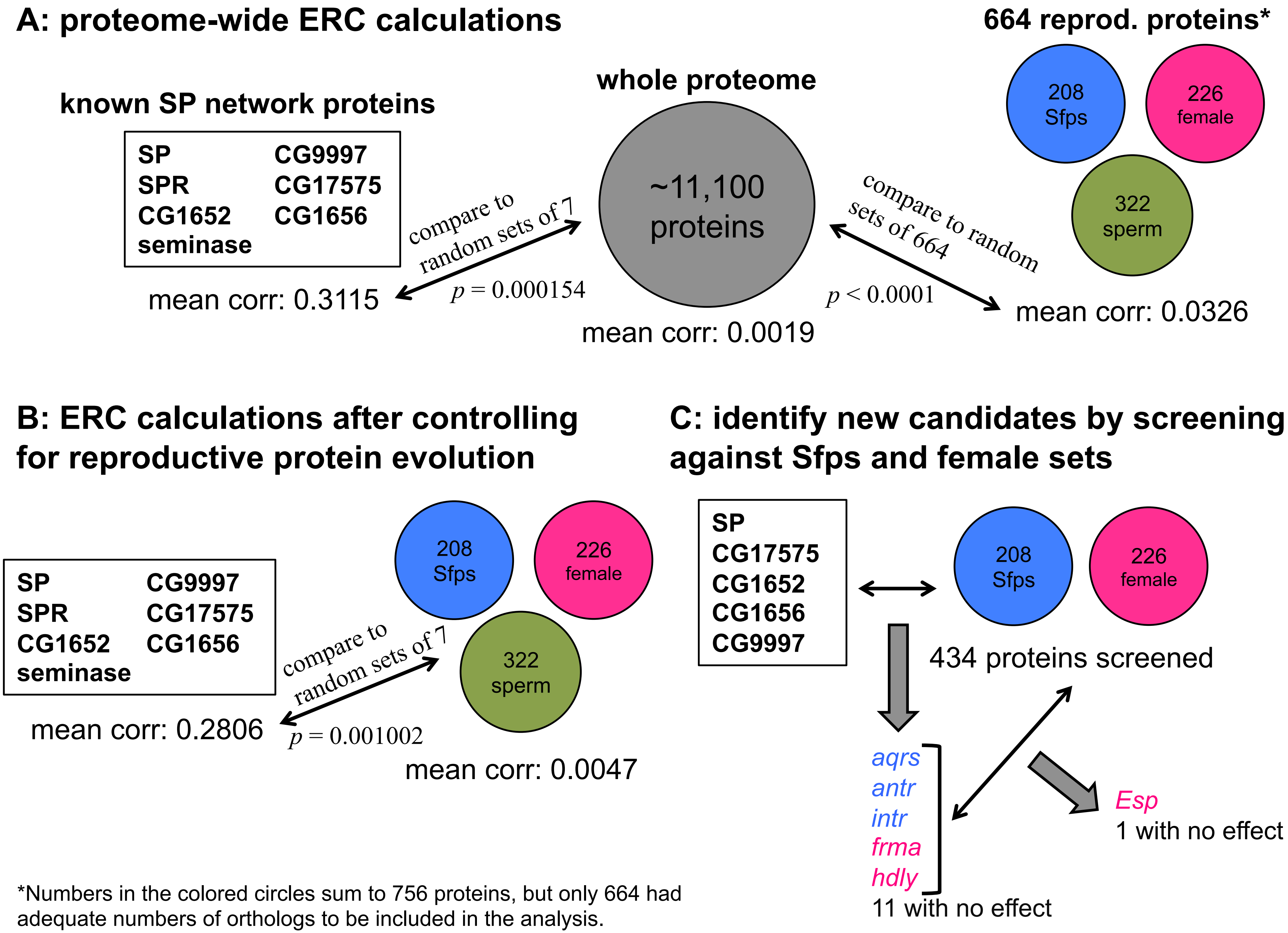

*Numbers in the colored circles sum to 756 proteins, but only 664 had adequate numbers of orthologs to be included in the analysis.
