# Supplementary figures and images for: Evolutionary Rate Covariation Identifies New Members of a Protein Network Required for Drosophila melanogaster Female Post-Mating Responses
Source: PLoS Genet. 2014 Jan 16;10(1):e1004108. doi: 10.1371/journal.pgen.1004108 (PMC3894160; doi:10.1371/journal.pgen.1004108)

***SPR***

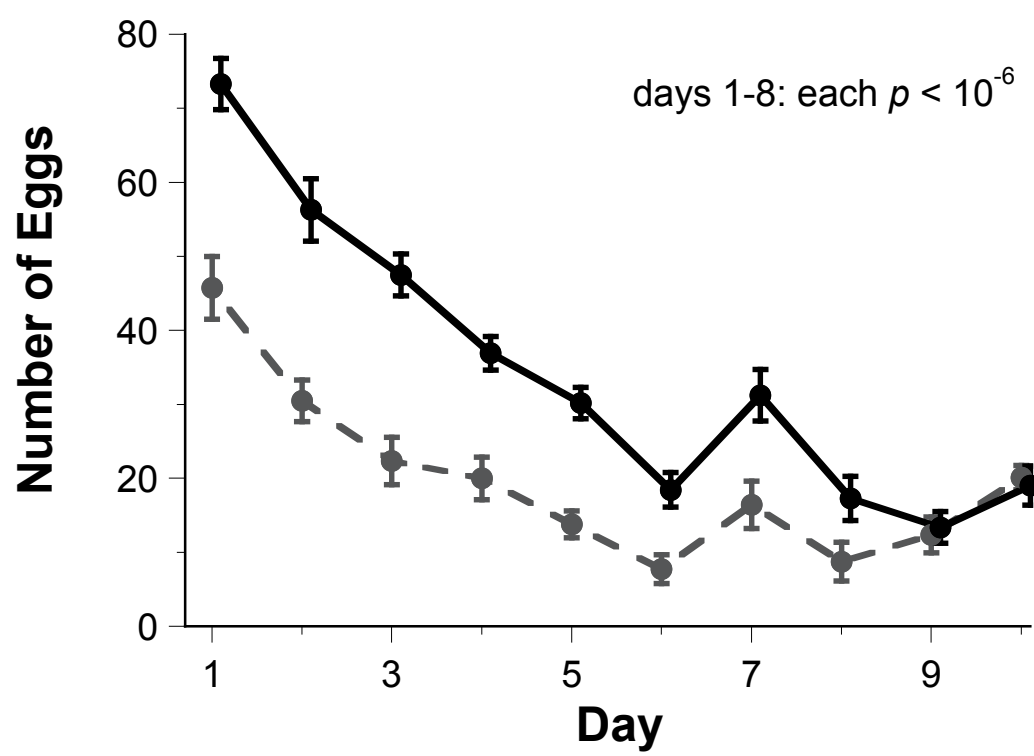

Supplement: Figure S2 — Fertility assay for females knocked down for SPR. This graph depicts the mean (± SE) number of eggs laid on each day of a 10-day fertility assay involving females knocked down for SPR (KD, dashed line; n = 16) and their controls (cont, solid line; n = 23). As previously reported, we observed a significant effect of knockdown on overall fertility (p<10−6), as well as significant differences on days 1–8 of the assay. Control data points are offset horizontally from knockdown data points to facilitate comparison, but all flies in each experiment were transferred from one vial to the next at the same time each day. These data are from one representative biological replicate. (PDF) [file pgen.1004108.s002.pdf]

**aquarius (CG14061)**

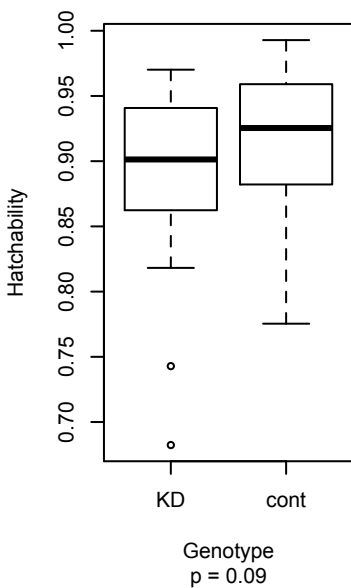

**antares (CG30488)**

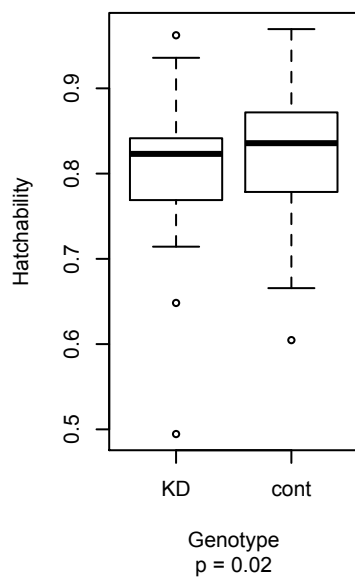

**intrepid (CG12558)**

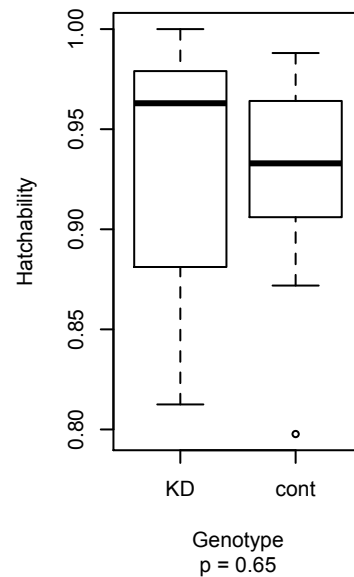

**fra mauro (CG3239)**

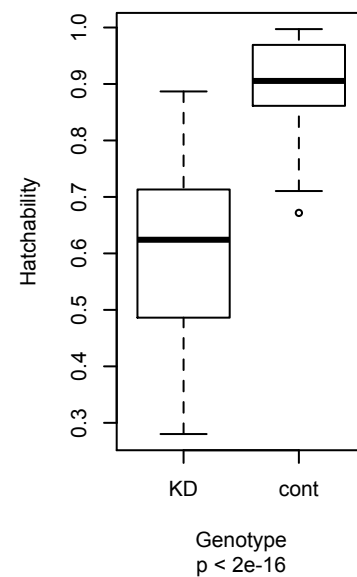

**hadley (CG5630)**

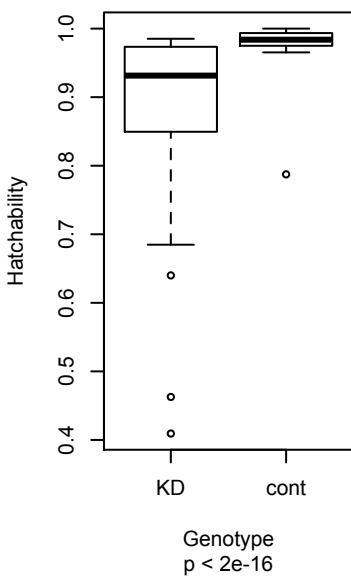

**Esp**

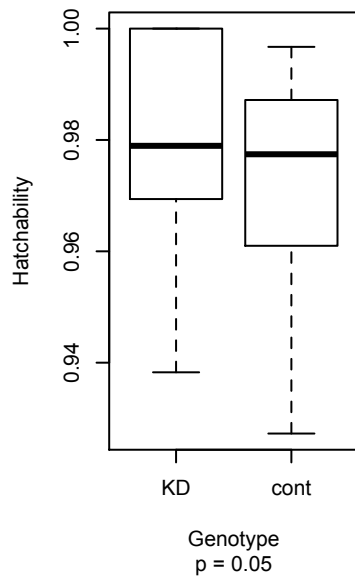

**SPR**

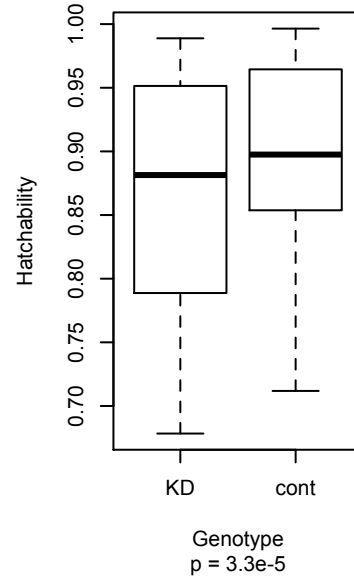

Supplement: Figure S3 — Overall rates of egg hatchability during 10-day fertility experiments. Each boxplot shows the distribution of egg hatchability rates for matings involving knockdown (KD) or control (cont) flies for each candidate gene. The thick black line represents the median rate of egg hatching across the entire 10-day assay; thin lines indicate the first and third quartiles; dots indicate outliers that lie further beyond the edge of box than 1.5× the interquartile range. P-values below each graph indicate results from statistical testing; after Bonferroni correction, p<0.0083 are considered significant. These data come from the experiments depicted in Figure 2. (PDF) [file pgen.1004108.s003.pdf]

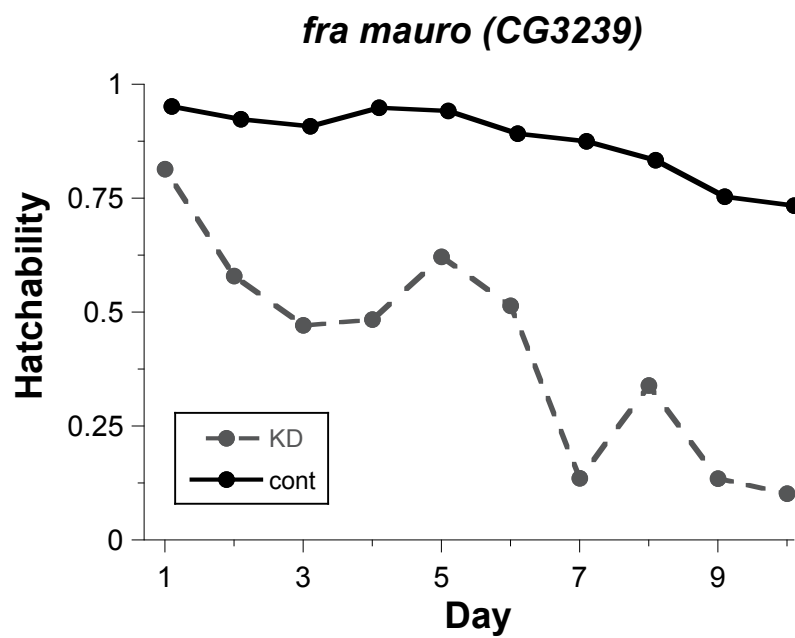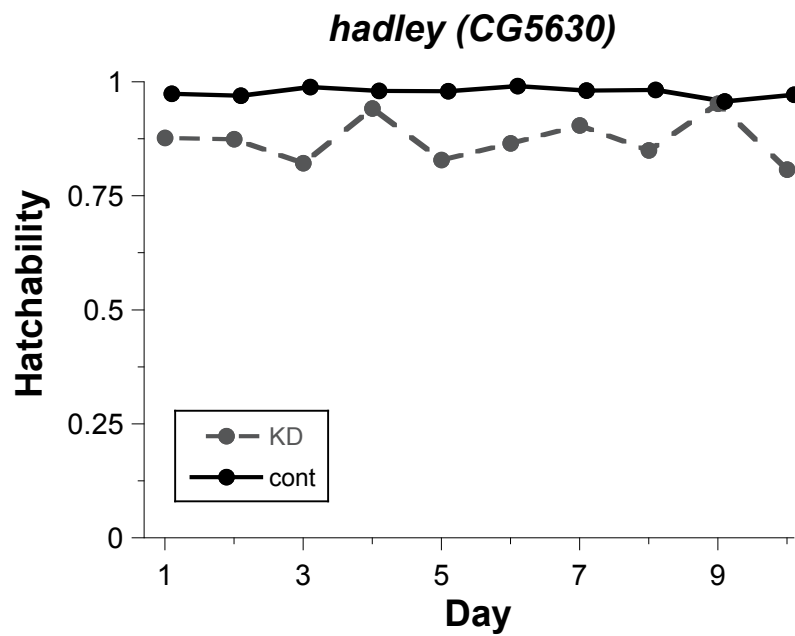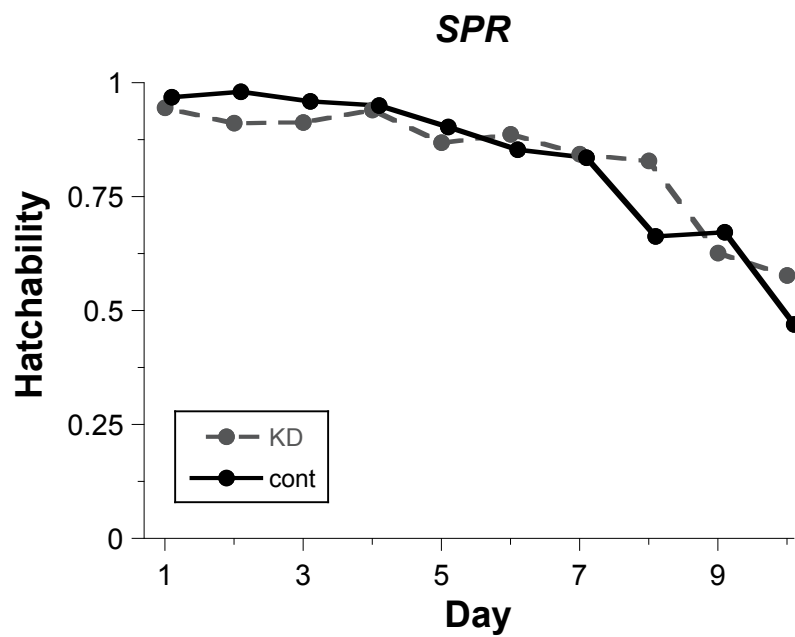

Supplement: Figure S4 — Day-by-day hatchability for female genes fra mauro (CG3239), hadley (CG5630) and SPR. Each point represents the total proportion of all eggs laid by all knockdown (KD) or control (cont) females that hatched on a given day during a 10-day fertility assay. These data come from the experiments depicted in Figure 2. (PDF) [file pgen.1004108.s004.pdf]

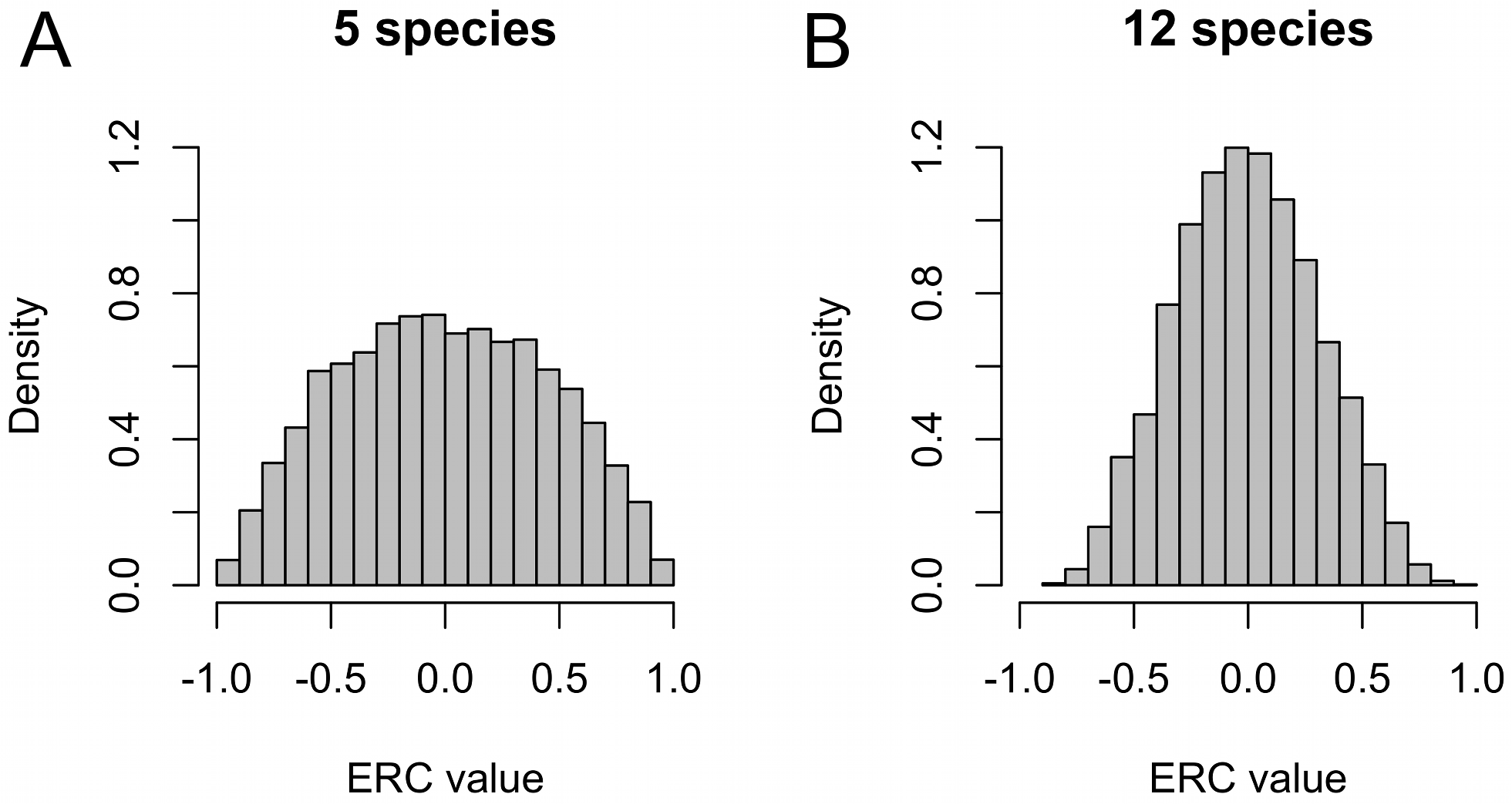

Supplement: Figure S6 — ERC values are more tightly distributed when more species are available for analysis. For each graph, 10,000 pairs of proteins were chosen randomly from the entire D. melanogaster proteome. ERC values were calculated using protein sequences from either (A) five closely related species (D. melanogaster, simulans, sechellia, yakuba and erecta) or (B) all 12 fully sequenced species of Drosophila [61]. (TIFF) [file pgen.1004108.s006.tif]

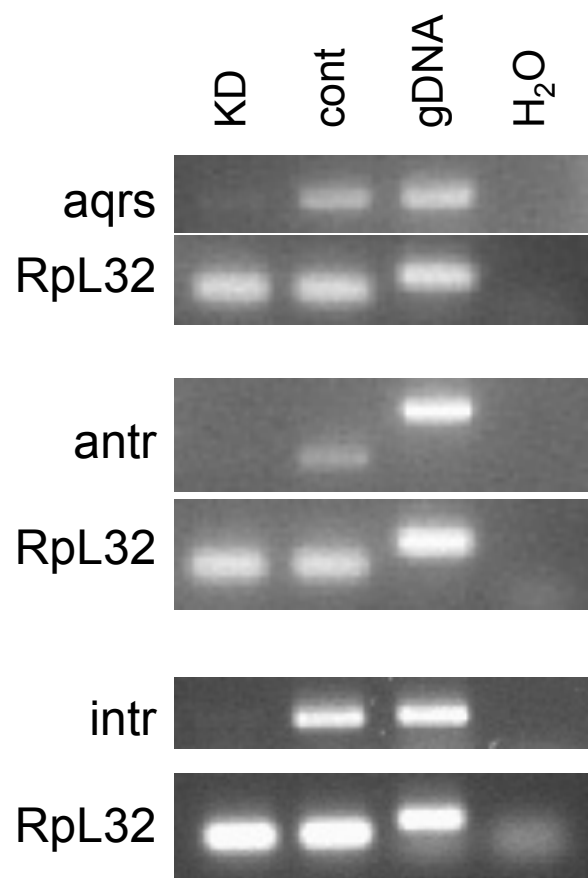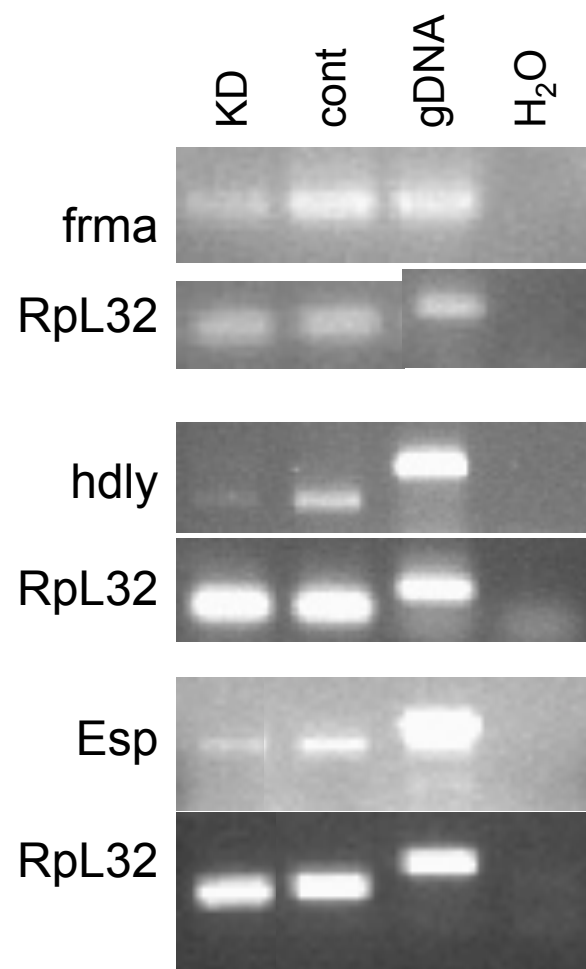

Supplement: Figure S7 — RT-PCR results verify RNAi knockdown for positive ERC candidate genes. Each gel shows PCR amplicons from reactions performed with a template of: cDNA synthesized from either knockdown (KD) or control (cont) flies of the appropriate sex, D. melanogaster genomic DNA (gDNA), or, as a negative control, water (H2O). In all cases, flies knocked down for a candidate gene showed either complete/near-complete (aqrs, antr, intr, hdly) or partial (frma) knockdown. When possible, RT-PCR primers were designed so that multiple exons would be amplified, resulting in larger products when gDNA was used as a template. (PDF) [file pgen.1004108.s007.pdf]
