# Supplementary material for: Evolutionary Rate Covariation Identifies New Members of a Protein Network Required for Drosophila melanogaster Female Post-Mating Responses
Source: PLoS Genet. 2014 Jan 16;10(1):e1004108. doi: 10.1371/journal.pgen.1004108 (PMC3894160; doi:10.1371/journal.pgen.1004108)
Supplement: Figure S5 — Alignment of protein sequences obtained by translating the 5′ untranslated region and the annotated coding region of the fra mauro gene in 12 Drosophila species. (PDF) [file pgen.1004108.s005.pdf]

```
 12 728
dmel         -----MHFKL RL-----LEL ---IV--GLL VVQVKSHPQ- ---------- ---ADPCQNF
dsec         -----MHLKL RL-----LEL ---FV--GFL LVQVKSRPQ- ---------- ---ANPCQNF
dsim         -----MHLKL RL-----LEL ---IV--GLL LVQVKSRPQ- ---------- ---ANPCQNF
dyak         -----MHCKR RL-----VVL ---IV--GLL LVQVKSHPQ- ---------- ---ADPCQNF
dere         -----MHFKR RL-----VVL ---IA--GLL LGQVKSHPQ- ---------- ---ADPCQNF
dana         -----MSFDL RL-----LLI ---ILLPGWA RPQSQPQPQP QLPDHVTRHM NRNVDPCRDF
dper         -----MRVDV QL-----LLM TTAIL--SFP SPCLCRRAQ- -LPSSMRRHM NRLADPCKDF
dpse         -----MRVDV QL-----LLM TTAIL--SFP SPCLCRRAQ- -LPSSLRRHM NRLADPCKDF
dwil         -----SSKLL SL-----LEL PKSIS----- ---LHIDPK- ---------- ---IDPCEDF
dgri         -----M-CKL QL-----LLL ----M--RLV LAILASDGW- --PGYLQLHM NASADPCLDF
dmoj         MFNVRELCKL QLWLPLGLSL ---CLSQCLW RPILASHAR- -LPVYIQRHM NASADACVDF
dvir         ------MCKL QS-----LLL ---SI--FVS IVVSNASQR- -LPGYLVRHM NASADACVDF

             YKVACGNWSA SHATDSYESF MDRLDYNYQE KLADLLDN-- -EREDDEPHF LQQLRNFYTA
             YKVACGNWSS SHATDSYQSF MDQLDYNYQE KLADLLDN-- -EREDDEPHF VQQLRAFYTA
             YKVACGNWSS SHETDSYQSF MDQLDYNYQE KLADLLDN-- -EREDDEPHF VQQLRAFYTA
             YGVACGNWSS IHASDSYQSV LGQLDYNYQA KLADLLDT-- -DRGEDEPHS VQQLRYFYTA
             YEVACGNWSS TEAT----SL LGQLDYNYQE KLADLLDT-- -EEKDDEPRF LQQLRYFYAA
             YKHACGKWAS AHAGMGYRSA VGQLDFNYHE QLADLLEQ-P LTADQDEPRF VGLVRDYYAA
             YEYACGNWMW THQGRPYRSL LEQLDHVYHG KLAKLLDQ-P PPQAAKEPRF VRMLRDYYTA
             YEYACGNWMW THQGRPYRSL LEQLDHVYHG KLAKLLDQ-P PPPAAKEPRF VRMLRDYYTA
             YNFACGNWAN VYRDRSYRSQ VDKLDYVFNR RLADLLEEEE EYNGKQNPYF VQLLKTNYNS
             YEHACGGWEE AHVTDAYSSQ LEQLDHVYHE QLASLLEQIT GAPSDGQPRF VELLRSSYAA
             YEHACGGWSL AHADDVYASQ LEQLDYEYHA SLAELLEQ-- -QPGPQEPRF VQLLRDSYVA
             YEHACGGWSQ AHAADAYNSQ LEQLDYMYHE RLALLLDQ-- -PPSPGQPRF VQLLRDSYAA

             CRKPLSQ-DQ VLRILEHLIV MENIQNEELS ---------- ----VGLTAA FRLQVLIDLN
             CRKPLSQ-DQ VLRILEHLIA MDNIQTEQLT ---------- ----VGLTAA FRLKVLPDIN
             CRKPLSQ-DQ VLRILEHLIA MDNIQTEQLT ---------- ----VGLTAA FRLKVLPDIN
             CRRPLSQ-DQ VLRSLEQLIA LEHIQFEEMT ---------- ----VGLTAA FRLEVIANIN
             CRRPLTQ-DQ VLRTLEQLIV LADIQFEELT ---------- ----VGLTAA FRLEVLADIN
             CRANHTV-VQ AMEVLARIIR KPSTSLEDLT ---------- ----VGLNTA FRVNILLDLH
             CRTPLEA-TP SAEFVLKLCG MAEVDGNELA ---------- ----VALTAL FQTDVLLEIA
             CRTPLEA-TP SAEFVLKLCG MAEVDGNELA ---------- ----VALTAL FQTDVLLEIT
             CRKLSRKTYK PDKFVQFLIN WSGKNFLNEP ---------N WERLHSVLMN FGLGQVLGKH
             CRDQQAG-YD AAQFVRWLRD LSGISMTSED ND-------N WSQVVSLLKG YGVAALQQFE
             CRRLDKR-YD AAQFVRWLAE WSRGETESET ESASASERGN GSALIHLLKT YGLGELLQYE
             CRALNER-YD VGRFVRWLRE FSHVDMETKE N--------D WRPLLRLIKN YGLPTMWQYE

             DSNT------ ----YDIW-K QLMSHRKHWD P-------NT TN--REPLTR EMFDKLWASL
             DSNT------ ----YDIW-K QLLSNGEHWD T-------HT TN--REPLTR EIFDKLWANM
             DSNT------ ----YDIW-K QLLSNREHWD T-------HT TN--REPLTR EIFDKLWANM
             HYNK------ ----YEIW-K LLLARQEHWD P-------NT TN--RESLTR DQFDELWVRM
             SDNK------ ----HEIW-K QLLSRRKDWD T-------NT TN--REPLTR DKFDELWASL
             GSEM------ ----TELWVT LILRHIEDWD E-------RE TR--RDAMTR GEFDRLWEHF
             DSNL------ ----AAVWAQ LILRPSAEGD R-------NA SL--HQPLSR EQFDQLWRRL
             DSNL------ ----ATVWAQ LILRPSAEGD R-------NA SL--HQPLSR EQFDQLWRRL
             LNTL------ ---DIDKY-R WLTHIQPLWP QFWQENNEET QTVILQPLRG HQFQRLFQKL
             MEMQ----PE SRSDAELW-- -LLQLQLPWP HSQAAATRNN CS--LSLLTR SRFRQLYREL
             NEEE-EEEAE AEALSDAE-Q RLLQLQLPWP N-PEDYLSDA GD--YEMLTH ASFRQLYREL
             NERKPLSKSL KLKDAELW-- -LLQLQPPWP VLPAELLDD- ----FQPLNR TRFRQLYHAL
```

```
PKIPFPFKEY YWRELSELEE KIM------- -SYGSEDDGF DSGDL----- --VTRIPPFW
PKMPFLFKEY YWHEMSELED KIM------- -SYGIKDDAS ARGDL----- --VTGIPSFW
PKVPFLFKEY YWHEMSKLED KIM------- -SYGIKDDAS ARGDL----- --VTGIPSFW
PKIPFPEKED FWYQVSQLEA ELT------- -SIGSEKDEV PRDEL----- --VTWIPSFW
PKKRSADKDL FWYQVSTLED EIM------- -SFGREDDGL PSDDL----- --VTWTPTSW
PAAELEDKES FWRNVTTVEE FLL------- -EHGEGEELA V--------- --LKGPPAFW
P-APEETPDE VWPKVKELEE AIT------- -AKVEKDALG WLPDTTTDNA GTLFPVPAYW
P-APEETPDE VWPKVKELEE AIT------- -AKVEKDGLG WLPDTTTYNA GTLFPVPAYW
H-LNLDNRTL -WRSIKHLEK QIT------- -QIQQDDIGT ES-------- --VISLPVNW
QPLGVPEGKL -WQQIKQLEK QLRLLWLLLH PSVVLDDASG VEVDL----- -------TRW
QPLGVAERRL -WKQLHRLEQ QLC------- -RCAQQQEQE QE-------- --QMRPLMLW
QPLGVPQGKL -WLQVKQLEK KLL------- -ICGIRELFG ELRESEHDEE HVVMPSAFYW

MMPW--PNGN ITYE------ NLSQMAHWLD IKANEF---- -ILTYIYMRL KLVAEGVSTE
MMPW--PNGN ITYE------ NLFEIAHFLD LKTDEF---- -IVVYIYMRL KLVPEGLSKE
MMPW--PNGN ITYE------ NLFEIAHLLD LKTDEF---- -IVVYIYMRL KLVPEGLSKE
MLPW--PNRY LTYR------ DLYSVARLLA LRTPQF---- -MLTYMYLRL KLLPEGLSKD
MLPW--PHRN LTYR------ NLYQVAYLLK IQTPGF---- -MLTYIYLRL RLVPEGRSRE
MLPW--PARL PDLW------ HFKRMLMVLT IHPAQF---- -LLRYIYLRL SLIPDPASCQ
MLPW--PTFE TGLS------ YVAHLSQYLA SQPPSLPPSL PAASFAAPRW RAGARGAS-P
MMPW--PTFE TGLS------ YVTHLSQYLA SQPPRF---- -LLHYLLLRS LH--RDGERA
ILPLSRNFSA PTEK------ YLQRLAHLLN QQSSGL---- -LAPYLLLRL QHRLEYSPEV
LLPL--PATT ESVEYLG-ET YLRGVSLLLA AQPPSL---- -VARYLQLRL LHQLELQPVP
LLPL--PAAS ARPSATRRPA YLSCVSALLA EQPVSL---- -LAAYLQLRL LRLLQQLPAP
LQLLEMPELL TTLQLQP--- HVRCASDMLA AEPAPF---- -VARYLQLRL LHKLDILPAP

SWHIDRDQCA EQSRQILSHP AAWLVEKNH- P---RLKEEP VLQDIFAELK QRFGQKLLAN
SWHIDRDQCA EQSRQMLSHP AVWLVEKNH- P---RLKEEP VLQDIFEELK HRFGQKLLAN
SWHIDRDQCA EQSTQILSHP AVWLVETTP- P---RLKEEP VLQDIFEELK HRFGQKLLAN
SWLMDRDQCA EQSRQILSHP AVWLIEQNH- P---RLQEEP MLLDIFEELK QRFGQKLRAN
SWLIDRDECA EQSRQILSHP AAWLMEKNH- P---RLQEEP VLQNIFEELK QRFGQKLRAN
PWAIESNDCA LQARELLGHP VVWLMEQHH- P---RLREDP VLQGLFGELK HRFGQKLRAN
AWS-----AL PRVGLFITHA AVWLVEAAP- P--------- ---------- ----------
PWSFTRLECA AQGRIFITHA AVWLVEQHH- P---RLQANG TLQSLFGELK QRFGLKLKAN
SPRFSRLPCS TQTRQFLTHA AVWLMASSE- N---FDSWDF LMQKLFTDIK QEFRLELIKN
PLAFGRQQCA AQSRQLLTHA VVWLMQEAQL PQLQRQQMRH SVQQLFAKLR RQFELKLLAN
AF--GRQQCA AQSRQLLTHA AVWLLQQQV- PHAQRHHTND TMHQLFEQLR QQFKLQLQVN
AF--GRQECA AQSRQLLTHA AVWLLEQQQ- PLEQRQLTNA TMHKLFEQLR HRFELRLLDN

RNNFTRSTQH FLLGKLKRMR LRLSILPRNS SAQSMVRRIE RHYRDVHMNA SDYFGNLHIG
RNNFTRSTQH FLLGKLKRMR LRLSILPRNC TAQSMVRRIE RHYRDVHMNA SDYFGNLNIG
RNNFTPHSTS FWSKK-KRMR LRLSILPRNC SAQSMVRRIE RHYRDVHMNA SDYFGNLNIG
RNNFTRRTQQ FLVRKLKRMR LRLSILPRNA SALNMGQLID GHYRHVHMNA SDYFGNLYIG
RNNFTRRTQR HLMSKLKRMR LRLSILPRNS SAQYMVRRID WHYRDVRMNA SDYFGNLYIG
RNQFSARVQA FLLAKLDRML LRLSVLPQRG ---DKVSAIA EHYQHLRLNA SDYFGNMLQL
------PPAA LLLEKLERMS LRLSILPSTE AVESVEERVE QHYKQLRINA TDYFGNLLAV
RNKFSAATQR FLLEKLERMS LRLSILPSAE AVESVEERVE QHYKQLRINA TDYFGNLLAV
RNHFSKESQQ FLYDKLDKMR LRLSVLPSGV RGSNLEQLLL KYYGDLTLNN SDYYGNLLKL
RNQFDAATQR FLLDKLRRMQ LRVGVLPPTG TANEHRLQLE THYAALQLNA SDYYGNLRAL
RNRFDNATQS FLLQKLQRMR LRVGVLP-SG SPEQQQQQLE EHYAQLQLRA NDYYGNLLAL
RNQFRPSTQR FLLEKLKRMR LRVGVLP-LG SAEQQQQTLE AHYAQLQLNA SDYYGNLLTM
```

```
LNHSRSHKKY AQLWAIVFGR QLIPSRISKS DLYPTQVRGY GTYASAFYIV KQNMLIVPLS
LNHSRSQRDY PELWAIVFG- -------PRV DSKPNLQERF VPHPSAW--- --NMLIVPLS
LNHSRSQRDY PELWAIVFGH EWIPSRTSKS ALYRTQVHGY GTFASAFYIV KQNMLIVPLS
LNHSRPQFNT L--------- --ASSRSPRS VLLPIQMHEY GTFASPFYIT EKNMLIVPLS
LNHSRPQLEN S-LWSVVFGR TLMPSRSSRS DLYPIQ-GNY GTFASPFYIT QQNILIVPLS
LDHHEAAGKR S--------- ---PSFEERK GWLPIDRQNY GSFASPFFLP RANMLVVPLS
MEHSRGQAEN P--------- ------ATKY DLHPVKVHGY GSYASPFFMP MGNALLVPLS
MEHSRGQAEN P--------- ------ATKY DLHPVKVHGY GSYASPFFMP MGNALLVPLS
INHIDYWSSW S--------- ------RVSE DFYIVQPDDY GSFASPFYLP QSNEMILPLS
LQLIDNDRQK V--------- -------ATA DLYFLQPDGF GSYASPFFLP GRNVVLLPHS
LRQTQRWAAG A--------- ----EGEAAD GLFVVQSDGF GSYASCFFLF SRNLVVLPHS
LGQVERWAAA A--------A DRATHMETDN ELYLLQSDGF GSYASPFFLP DSNLVILPQS

LLEPPFYTHG QPSILTYSAL GFILGHELSH GFDSE-GMTF SSHGVGSSAV DRE-LDRNPR
LLEPPFYTHG QPAILTYSSL GFILGHELSH GFDSQ-GMTF SSNGVDNSAV DTE-LEWNPR
LLEPPFYTHG QPAILTYSSL GFILGHELSH GFDSE-GMTF SSNGVDNSAV DTE-LEWNPR
LLEPPLYAHD QPPILTYSSL GFILAHELSH GFDSE-GVTY SADGVASRAV YRE-IGRNPR
LLEPPLYSPG QPPLLTYSSL GFILGHELSH GFDSE-GVTF SANGMMSRTV NRE-IEGNTR
LLDSRLYERG QADLLTYSTL GFILGHELTH GFGFH-EVGR SQRGHLHPGV ARE-LGRNQA
L-----WRRR STGLSSASSL GFILGHELSH GFAPV-DVHF DAFGQPNRSK SLS-MLTSRR
LLEAPLYRPE QRQVLTYSSL GFILGHELSH GFAPV-DVHF DAFGQPNRSK SLS-MLTSRR
LLGLSVYRAN QSNLWTYSSL GFLIGHELSH GFTPNFAFLY DSQGNRLIGK ----LETTAR
LFGGHLYWPD QAAIYRYSGL GFLIAHELSH GFAPS-DVHY DGCGNEANAQ QQQRLLTNRH
LLAANLYRSH QPKVFTHSGL GFLLAHELSH GFDLS-GVVY DGRGKLASRQ QRQRLSSNAR
LLGANLYRPH QAEVYTHSAL GFLLAHELSH GFAPT-DVFY DGRGNKARGQ QKLRLLVNRR

FQQELGCLRR RFGRKRY-EK FADASGLELA YSAYFDT--- --AQTDHKRN RSAEELVTQK
FQQKLDCLDR RFSSRRY-EK FADASGLELA YSAYFDT--- --AQTDHKRK RSAEELVAQK
FQQKLDCLDR RFSNRRY-EK FADASGLELA YSAYFDT--- --AQTDHKRK RSAEELVAQK
FQHEVSCLDR KFGSRRY-EK FADASGLELA YSAYFDT--- --AQTDRKRH RSTDNLLSQQ
LQHEFRCLYR KFGSRWY-EK FADASGLELA YSAYFDI--- --AQTDRKRN RSTEHLVTQK
FRRAKDCVAD RFGPKHSEEK LADAMGLELS YSAYFDE--- --AETDRSRN RSGTGTPEQR
FLSQVLCLHR RHGMFIADEK FADLNGLDLA YGAYFEA--- --------GG GPGRCTPAQK
FLSQVLCLHR RHGMFIADEK FADLNGLDLA YGAYFEA--- --------GG GPGRCTPAQK
FLEKGKCLKD LYGDMAD-EK FADLMGLTMA FKAYA----- --------KL ESQPITKRER
FGKEMRCLRR RHGQMAD-EK FADINGIALA YDSYF----- --------AG HPASTAAKQQ
FANQKTCLKR RHAEIAD-EK FADLNGLSLA YDSYF-T--- --AHCQHKEA TGARCGTAVQ
FSSHRLCLLR RHDQMAD-EK FADVNGLYLA YDNYFKSNNA TPSSATADNG NSSEGRHTVQ

QQFFHNFAQF FCSDKE---L --LQAHDHGS DRKRVNDAVA HFEPFREAFS CG---PS--P
QQFFLNFAQY FCSNME---L D-KEAPTHGS DRKRVNDAVA HFEPFREAFS CG---PS--P
QQFFLNFAQF FCSNKE---L D-KEAPDHGS DRKRVNDAVA HFEPFREAFS CG---PS--P
QQFFLNFARF FCSDGE---L --SEESDHGS DRKRVNDAVA HFKPFREAFH CG---TS--H
QQFFLNFAQF FCSDEDYEQL --LEQSAYGS DRKRVNDAVA HFKPFQEAFS CG---TS--H
QLFFLNLAQF FCSSERHSDW ----RSYHGS DRRRVNDAMK AFPPFQEAFG CP---AALRP
QLFFFNFAQF FCSDDR--NL --EDSEEHGS DRRRVNDAMA SFDPFRQAYG CK---SVRRG
QLFFFNFAQF FCSDDR--NL --EDSEEHGS DRKRVNDAMA SFDPFRQAYG CK---SVRRG
QQFFLNFAQF FCEAKD--EL ESVTNEEHGG NRERVNEVIE NLSTFRQSFH CEGLVKNKGG
QLFFLNFAQF FCQDEQQLEE DVEDSNQHGD SRQRVNDAVA SSESFALAFS CDRQKEM--K
QHFFLNFAQF FCRDD----L QPEDTSEHGS SRQRVNDAVA SSQHFARAFG CE---RS-RH
QHFFLNFAQF FCQDDP--DL --EDSSLHGG SRQRVNDAVA SSKPFARAFG CE---WT--Q
```

RRRQCRLY
RRRQCRLY
RRRQCRLY
RRRHCRLF
RRRQCRLF
KRKPCRLY
SRTRCQLY
SRTRCQLY
PSRKCKLY
RRRICQLY
KHPLCQLY
IRNTCQLY
