# Supplementary material for: Evolutionary Rate Covariation Identifies New Members of a Protein Network Required for Drosophila melanogaster Female Post-Mating Responses
Source: PLoS Genet. 2014 Jan 16;10(1):e1004108. doi: 10.1371/journal.pgen.1004108 (PMC3894160; doi:10.1371/journal.pgen.1004108)
Supplement: Protocol S1 — Supporting methods. (PDF) [file pgen.1004108.s008.pdf]

Protocol S1. Supporting methods

RT-PCR

To evaluate knockdown, we isolated RNA from whole flies (when *tubulin*-GAL4 was used as the driver) or from dissected reproductive tissues (when *Send1*-GAL4 or *ovulin*-GAL4 was used as the driver) using the TRIzol reagent (Invitrogen). Genomic DNA was removed by treating 1 ug RNA with 1 unit of RQ1 DNase (Promega) according to the manufacturer's instructions. We then used ~0.35 ug of DNase-treated RNA per sample to synthesize cDNA, using SmartScribe reverse transcriptase (Clontech). The resulting cDNA was diluted 10-fold, and 1 uL was used per PCR reaction to test for gene expression between knockdown and control flies. Primers for PCR were designed with Primer3 (http://frodo.wi.mit.edu/) to amplify ~350 bp cDNA fragments. When possible, primers were designed so that genomic DNA would produce either a larger product (i.e., because it would contain a small intron) or no product (i.e., because it would contain a large intron). As a positive control for RT-PCR, we amplified an intron-containing region of the *RpL32* gene, the primers for which were designed so that genomic DNA would produce a larger product. In no case was gDNA contamination observed. In general, PCR products and expression patterns could be readily discerned with 30 cycles of amplification; for lowly expressed genes, we performed 35 cycles of PCR.

Initial screen for 4-day remating receptivity

Briefly, knockdown males and their controls were mated to wild-type (lab strain Canton S) females, and the females were given an opportunity to remate 4 days later with a Canton S male. Knockdown females and their controls were mated to Canton S males, and then given an opportunity to remate 4 days later with another Canton S male. Females were aged individually between mating trials. Any candidate gene that had a significant effect on remating receptivity at 4 days after an initial mating was then screened for an effect on remating receptivity at 1 day after an initial mating [1]. Differences in the remating rates between treatments were analyzed by Fisher's exact test. We also used the 4-day assay to evaluate receptivity in crosses involving additional RNAi lines (Table S3).

Measurements of fertility, fecundity and egg hatchability

Genes that showed a significant effect on female remating receptivity were then screened for effects on fertility, fecundity and egg hatchability using standard assays [1,2] with two slight modifications as described here. First, for assaying female-expressed genes, knockdown or control females were mated to Canton S males, and the males were then removed. Second, for measuring hatchability, we counted the number of pupal cases observed in each vial after adults had eclosed. We took this measure of progeny count and divided it by the number of eggs counted to calculate egg hatchability. In cases where progeny exceeded the number of counted eggs, we inferred that an egg had been missed in counting, e.g., because of females' propensity to lay eggs into crevices and bubbles in food media. In these cases, the number of eggs was adjusted upward to the number of observed progeny, reflecting the fact that each progeny must be produced from an egg. Thus, egg hatchability ranged from 0 to 1.

Statistical differences between treatments were determined as previously described [2]. Briefly, fecundity differences between treatments across the entire 10-day assay were determined by repeated measures analysis using a Poisson mixed-effects model as previously described. Statistical differences in egg-hatchability between treatments over the course the assay were determined in the same way, except using a Binomial model. These data were also analyzed with non-parametric Wilcoxon rank-sum tests, which gave equivalent results. To determine the effect of male or female genotype on fecundity on each day of an assay, we used a repeated measures analysis with a Poisson-mixed effects model, as described in ref. [2].

In assays to replicate phenotypes in additional RNAi lines (Table S3), we measured long-term fertility by transferring mated females to a new vial 3 days after an initial mating. Twenty-four hours later, we conducted the 4-day receptivity test as described above. Both males and females were then immediately discarded, and eggs laid over the prior 24-hour period were allowed to develop. We then counted the number of pupal cases observed in each vial after adults had eclosed and analyzed differences in progeny number between knockdown and control matings by Student's t-tests.

Sperm counting

SP and the previously described SP network proteins are required for the release of sperm from storage in the seminal receptacle [1,3]. We counted the number of sperm stored in the SR at two time points: 2 hours after mating, and 10 days after mating. The early time point provided a baseline for the amount of sperm initially stored, and the late time point allowed us to assess the rate of sperm release from storage. Females mated to knockdown or control males were flash frozen at each time point. We then followed standard procedures [2,3] to dissect female reproductive tracts, unravel the SR, and stain for sperm nuclei with 0.2% orcein in 50% acetic acid. Coded slides were counted under 1000x magnification on an Olympus BX41 microscope. A subset of samples was counted twice to estimate the percent concordance in counting. For each experiment, the median percent concordance was >92 percent. Data were analyzed with a two-tailed Student's t-test [as in ref. 2] and with non-parametric Wilcoxon rank-sum tests, which gave equivalent results.

References

1. Ravi Ram K, Wolfner MF (2007) Sustained post-mating response in *D. melanogaster* requires multiple seminal fluid proteins. Plos Genetics 3: e238.
2. LaFlamme BA, Ram KR, Wolfner MF (2012) The *Drosophila melanogaster* seminal fluid protease "seminase" regulates proteolytic and post-mating reproductive processes. Plos Genetics 8.
3. Avila FW, Ram KR, Qazi MCB, Wolfner MF (2010) Sex peptide is required for the efficient release of stored sperm in mated *Drosophila* females. Genetics 186: 595-600.
