# Supplementary material for: Evolutionary Rate Covariation Identifies New Members of a Protein Network Required for Drosophila melanogaster Female Post-Mating Responses
Source: PLoS Genet. 2014 Jan 16;10(1):e1004108. doi: 10.1371/journal.pgen.1004108 (PMC3894160; doi:10.1371/journal.pgen.1004108)
Supplement: Table S1 — RNAi lines, drivers and crosses used in this study. (PDF) [file pgen.1004108.s009.pdf]

Table S1. RNAi lines, drivers and crosses used in this study.

Gene	Sex	RNAi Line	Driver	Crosses used to generate flies**
CG34033	M	homemade TRiP line	tubulin-GAL4	KD: RNAi x tubulin-GAL4<br>cont: y v 1509 x tubulin-GAL4
CG11037	M	VDRC-KK transformant 108803	ovulin-GAL4*	KD: RNAi x ovulin-GAL4<br>cont: AttP x ovulin-GAL4
CG11977	M	VDRC-KK transformant 100463	ovulin-GAL4*	KD: RNAi x ovulin-GAL4<br>cont: AttP x ovulin-GAL4
CG14034	M	VDRC-KK transformant 100114	tubulin-GAL4	KD: RNAi x tubulin-GAL4<br>cont: RNAi x w1118
**CG14061 (aquarius)**	M	homemade TRiP line	tubulin-GAL4	KD: RNAi x tubulin-GAL4<br>cont: y v 1509 x tubulin-GAL4
CG2975	M	VDRC-GD transformant 2601	ovulin-GAL4	KD: RNAi x ovulin-GAL4<br>cont: AttP x ovulin-GAL4
**CG30488 (antares)**	M	VDRC-KK transformant 100513	tubulin-GAL4	KD: RNAi x tubulin-GAL4<br>cont: AttP x tubulin-GAL4
CG42326	M	VDRC-KK transformant 104709	tubulin-GAL4	KD: RNAi x tubulin-GAL4<br>cont: RNAi x w1118
**CG12558 (intrepid)**	M	VDRC-GD transformant 14844	tubulin-GAL4	KD: RNAi x tubulin-GAL4<br>cont: TM3 siblings of KD cross

CG13077	F	VDRC-KK transformant 105526	tubulin-GAL4	KD: RNAi x tubulin-GAL4<br>cont: AttP x tubulin-GAL4
CG3097	F	homemade TRiP line	tubulin-GAL4	KD: RNAi x tubulin-GAL4<br>cont: y v 1509 x tubulin-GAL4
**CG3239 (fra mauro)**	**F**	TRiP line Bloom stock 34558	tubulin-GAL4	KD: RNAi x tubulin-GAL4<br>cont: y v 1509 x tubulin-GAL4<br>(also attempted KD with Send1-GAL4, but saw no phenotype)
CG6910	F	VDRC-KK transformant 103766	Send1-GAL4; UAS-Dicer2	KD: RNAi x Send1-GAL4; UAS-Dicer2<br>cont: AttP x Send1-GAL4; UAS-Dicer2
CG42564	M	VDRC-KK transformant 101703	ovulin-GAL4*	KD: RNAi x ovulin-GAL4<br>cont: AttP x ovulin-GAL4
CG8420	M	VDRC-KK transformant 104679	ovulin-GAL4*	KD: RNAi x ovulin-GAL4<br>cont: AttP x ovulin-GAL4
**CG5630 (hadley)**	F	VDRC-GD transformant 52066	tubulin-GAL4	KD: RNAi x tubulin-GAL4<br>cont: RNAi x w1118
SPR (control)	F	VDRC-KK transformant 106804	tubulin-GAL4	KD: RNAi x tubulin-GAL4<br>cont: RNAi x w1118
CG30486	M	VDRC-KK transformant 107568	tubulin-GAL4	KD: RNAi x tubulin-GAL4<br>cont: AttP x tubulin-GAL4
CG34295	M	VDRC-KK transformant 109546	tubulin-GAL4	KD: RNAi x tubulin-GAL4<br>cont: AttP x tubulin-GAL4
sda	F	VDRC-KK transformant 100215	tubulin-GAL4	KD: RNAi x tubulin-GAL4<br>cont: AttP x tubulin-GAL4

**Esp (CG7005)**	F	VDRC-GD transformant 9795	tubulin-GAL4	KD: RNAi x tubulin-GAL4<br>cont: RNAi x w1118

* knockdown with tubulin-GAL4 caused pre-adult lethality

**We used four types of controls in our experiments:

-For most experiments with VDRC lines, we crossed the RNAi line and its genetic background control to the driver line. (The tubulin-GAL4 line had been backcrossed for 7 generations into a w1118 background that had itself been backcrossed for 8 generations.)

-For some experiments, we crossed the RNAi line to F7 tubulin-GAL4 (for KD flies) and to F8 w1118 (for controls).

-For crosses involving a TRiP RNAi line, we crossed the RNAi line and the y v 1509 genetic background control to the driver line.

-For crosses involving intrepid, controls were flies that carried the RNAi construct but received TM3, Sb instead of tubulin-GAL4

Additionally, crosses involving the following genes (VDRC stock number) did not produce detectable knockdown:
CG16713 (105067), CG4302 (100167), CG8586 (106597), Mtp (110414), vkg (106812)
