# Supplementary material for: Evolutionary Rate Covariation Identifies New Members of a Protein Network Required for Drosophila melanogaster Female Post-Mating Responses
Source: PLoS Genet. 2014 Jan 16;10(1):e1004108. doi: 10.1371/journal.pgen.1004108 (PMC3894160; doi:10.1371/journal.pgen.1004108)
Supplement: Table S2 — Genomic locations of SP network proteins in Drosophila melanogaster. (PDF) [file pgen.1004108.s010.pdf]

Table S2. Genomic locations of SP network proteins in *D. melanogaster*.

Gene	Chromosomal Location [coding strand]	Functional Class	Clear Evidence of Tandem Duplication
CG1652	2R:5,700,867..5,704,105 [+]	lectin	yes
CG1656	2R:5,704,805..5,706,040 [+]	lectin	yes
CG17575	2R:8,733,049..8,734,101 [-]	CRISP	no
antares	2R:8,734,353..8,735,509 [-]	CRISP	no
SP	3L:13,294,735..13,295,022 [+]	peptide hormone	n/a
seminase	3L:20,948,855..20,949,850 [-]	protease homolog	n/a
hadley	3R:16,740,018..16,754,935 [+]	unknown	n/a
Esp	3R:20,667,657..20,676,134 [+]	sulfate transporter	n/a
CG9997	3R:24,581,206..24,582,701 [-]	protease homolog	no
aquarius	3R:24,582,721..24,583,956 [-]	protease homolog	no
intrepid	3R:24,616,244..24,617,183 [+]	protease homolog	no
SPR	X:5,340,694..5,387,590 [+]	GPCR	n/a
fra mauro	X:5,388,073..5,390,082 [-]	neprilysin	n/a
