# Supplementary material for: Evolutionary Rate Covariation Identifies New Members of a Protein Network Required for Drosophila melanogaster Female Post-Mating Responses
Source: PLoS Genet. 2014 Jan 16;10(1):e1004108. doi: 10.1371/journal.pgen.1004108 (PMC3894160; doi:10.1371/journal.pgen.1004108)
Supplement: Table S3 — Measures of receptivity and fertility for additional RNAi lines for positive ERC candidate genes. (PDF) [file pgen.1004108.s011.pdf]

Table S3. Measures of receptivity and fertility for additional RNAi lines for positive ERC candidate genes.

Gene	Replicate RNAi Line	4-Day Receptivity	Day 4 Fertility (mean ± SE)
CG14061 (*aqrs*)	none available	n/a	n/a
CG30488 (*antr*)	VDRC-GD transformant 41281 (control for siRNA sequence and insertion site)<br>control flies: UAS x F_8 w^{1118}	KD: 20/26<br>cont: 0/24<br>$p < 0.0001$	KD: 0.3 ± 0.3<br>cont: 29.5 ± 2.3<br>$p < 0.0001$
CG12558 (*intr*)	none available	n/a	n/a
CG3239 (*frma*)	VDRC-KK transformant 102309 (control for siRNA sequence and insertion site)<br>control flies: AttP x F_7 *tub*-GAL4	KD: 5/25<br>cont: 4/24<br>$p = 1.00$	KD: 29.9 ± 3.3<br>cont: 44.4 ± 3.8<br>$p < 0.007$
CG5630 (*hdly*)	VDRC-GD transformant 52067 (control for UAS insertion site)<br>control flies: UAS x F_8 w^{1118}	KD: 6/32<br>cont: 1/33<br>p = 0.054	KD: 20.8 ± 3.5<br>cont: 48.4 ± 3.0<br>$p < 0.0001$
Esp	VDRC-GD transformant 9797 (control for UAS insertion site)<br>control flies: UAS x F_8 w^{1118}	KD: 13/29<br>cont: 3/18<br>$p = 0.0457$	KD: 22.4 ± 2.4<br>cont: 35.8 ± 2.7<br>$p < 0.0007$

*Knockdown of *frma* for line VDRC-KK 102309 was only partial, consistent with a less severe fertility phenotype. Other knockdown flies described above showed the following levels of knockdown: *antr* – near complete; *hdly* – near complete; *Esp* – complete.
