# Supplementary material for: Evolutionary Rate Covariation Identifies New Members of a Protein Network Required for Drosophila melanogaster Female Post-Mating Responses
Source: PLoS Genet. 2014 Jan 16;10(1):e1004108. doi: 10.1371/journal.pgen.1004108 (PMC3894160; doi:10.1371/journal.pgen.1004108)
Supplement: Text S1 — Supporting results text testing for whether the egg hatching defects observed in fra mauro or hadley females could be explained by reduced offspring viability. (PDF) [file pgen.1004108.s012.pdf]

Text S1. Supporting results for hatchability data

The decreased egg production by *frma* (*CG3239*) and *hdly* (*CG5630*) knockdown females (Figure 2), coupled with their increased remating receptivity (Table 1), strongly support these genes' importance for SP function in mated females. However, we also observed that eggs laid by these females showed decreased hatchability (Figures S3-S4), which could arise for several reasons. The hatchability phenotype could result from perturbed SP-mediated processes, as discussed in the main text. However, since these genes are female-expressed, it is also very possible that their knockdown could result in a maternal effect that impacts embryonic development or could cause production of eggs that cannot be fertilized efficiently. Alternatively, since some fraction of progeny from *frma* or *hdly* knockdown females receive both the UAS-RNAi construct and the tubulin-GAL4 driver, it is possible that knockdown of either gene could reduce egg-to-adult viability. To address this latter issue, we examined the frequency with which the tubulin-GAL4 driver was inherited in the following test crosses. (For simplicity, we show only the relevant chromosomes in these crosses; complete details of the RNAi lines used are given in Table S1.)

For fra mauro (CG3239):

Knockdown cross: UAS-RNAi / UAS-RNAi x tubulin-GAL4 / TM3, Sb
Control cross: + / + x tubulin-GAL4 / TM3, Sb

For hadley (CG5630):

Knockdown cross: UAS-RNAi / UAS-RNAi x tubulin-GAL4 / TM3, Sb
Control cross: AttP / AttP x tubulin-GAL4 / TM3, Sb

We compared the frequency of Sb^+ progeny between the knockdown and control crosses, as these progeny should be knocked down for the target gene. Knockdown of *hadley* caused no significant lethality: 49.5% (102/206) of progeny from the knockdown cross were Sb^+, while 50.9% (108/212) of progeny from the control cross were Sb^+ ($\chi^2 = 0.085$, 1 d.f., $p = 0.77$). However, knockdown of *fra mauro* caused significant lethality: only 34.3% (56/163) of progeny in the knockdown cross received Sb^+, while 49.8% (128/257) of progeny in the control cross were Sb^+ ($\chi^2 = 9.67$, 1 d.f., $p = 0.002$). This lethality manifested itself predominantly as a lack of knockdown male progeny:

Class	Count
Sb^+ females	46
Sb^+ males	10
Sb females	56
Sb males	51

Thus, ubiquitous knockdown of *fra mauro* during development causes partial lethality, particularly in male progeny.

We next evaluated whether the degree of partial lethality caused by knockdown of *fra mauro* in the experiment above could account for the amount of decreased hatchability observed in the egg-laying experiment (Figures S3-S4). Progeny produced in the egg-laying experiment result from this cross:

Wild-type males x $y\ w^{+}\ v\ /\ y^{+}\ w\ v^{+}$; + / +; UAS-RNAi $y^{+}\ v^{+}$ / *tub*-GAL4 w^{+}

Knockdown female parents in this cross have the RNAi construct and the tubulin-GAL4 driver in trans on chromosome III; the genetic distance between these two insertions is unknown. Assuming conservatively that the insertions are >50 map units apart, the expected fraction of progeny that would inherit a Y chromosome from the male parent and both the UAS-RNAi and the driver from the female parent is 1/8. Since the reduction in hatchability observed for *fra mauro* knockdown females was far greater than this proportion (Figures S3-S4), we conclude that lethality caused by the co-inheritance of UAS-RNAi and tubulin-GAL4 can explain only a minor fraction of the hatchability defect observed for *frma* knockdown females in the fertility assay. Thus, the hatchability defect likely reflects a problem associated with an SP-related process, a maternal effect of knockdown in the germline or associated follicle cells, or the reduced efficiency of fertilization of eggs of knockdown females.

Regardless of the cause of the hatchability defect, we emphasize that the phenotype of *frma* and *hdly* females that is of primary relevance to the SP network is their significantly reduced egg production (Figure 2).
